# Histone Deacetylase Inhibitors Enhance CD4 T Cell Susceptibility to NK Cell Killing but Reduce NK Cell Function

**DOI:** 10.1371/journal.ppat.1005782

**Published:** 2016-08-16

**Authors:** Matthew Pace, James Williams, Ayako Kurioka, Andrew B. Gerry, Bent Jakobsen, Paul Klenerman, Nneka Nwokolo, Julie Fox, Sarah Fidler, John Frater, CHERUB Investigators

**Affiliations:** 1 Peter Medawar Building for Pathogen Research, Nuffield Department of Medicine, Oxford, United Kingdom; 2 Institute for Emerging Infections, The Oxford Martin School, Oxford, United Kingdom; 3 Adaptimmune Ltd, Oxfordshire, United Kingdom; 4 Oxford National Institute of Health Research Biomedical Research Centre, Oxford, United Kingdom; 5 Chelsea and Westminster Hospital, London, United Kingdom; 6 Department of Genitourinary Medicine and Infectious Disease, Guys and St Thomas' NHS Trust, London, United Kingdom; 7 Division of Medicine, Wright Fleming Institute, Imperial College, London, United Kingdom; University of North Carolina at Chapel Hill, UNITED STATES

## Abstract

In the search for a cure for HIV-1 infection, histone deacetylase inhibitors (HDACi) are being investigated as activators of latently infected CD4 T cells to promote their targeting by cytotoxic T-lymphocytes (CTL). However, HDACi may also inhibit CTL function, suggesting different immunotherapy approaches may need to be explored. Here, we study the impact of different HDACi on both Natural Killer (NK) and CTL targeting of HIV-1 infected cells. We found HDACi down-regulated HLA class I expression independently of HIV-1 Nef which, without significantly compromising CTL function, led to enhanced targeting by NK cells. HDACi-treated HIV-1-infected CD4 T cells were also more effectively cleared than untreated controls during NK co-culture. However, HDACi impaired NK function, reducing degranulation and killing capacity. Depending on the HDACi and dose, this impairment could counteract the benefit gained by treating infected target cells. These data suggest that following HDACi-induced HLA class I down-regulation NK cells kill HIV-1-infected cells, although HDACi-mediated NK cell inhibition may negate this effect. Our data emphasize the importance of studying the effects of potential interventions on both targets and effectors.

## Author Summary

Antiretroviral therapy successfully controls HIV-1 viraemia and can restore life expectancy to within normal limits. However, antiretroviral therapy is not a cure as HIV-1 persists in a treatment-resistant latent reservoir. Therapy also comes with a high cost, side effects, and a lifetime commitment to pills. Therefore, there is growing interest in finding a cure. One proposed strategy is to use novel agents to stimulate HIV-1 transcription in latently-infected cells, after which the cells could be targeted and cleared by the immune system. Of current interest are the latency-reversing histone deacetylase inhibitors (HDACi), with CD8 T cells and NK cells potentially serving as the HIV-1 killing agents. However, previous studies suggested HDACi negatively affected CD8 T cell function, compromising their role as killers. Here we studied whether NK cells might serve as effective killers of HDACi treated CD4 T cells, especially as HDACi may induce HLA class I down-regulation. We found that although treating infected cells with HDACi resulted in HLA class I down-regulation and enhanced their visibility to NK cells, HDACi inhibited NK cell function overall, suggesting additional killing strategies will be required.

## Introduction

Antiretroviral therapy (ART) is capable of controlling viraemia in HIV-1-infected individuals to undetectable levels. However, ART is not a cure. A pool of latently infected cells persists, despite therapy, for the lifetime of the individual. One approach to target this latent reservoir is to activate latently-infected cells to stimulate virus production followed by cytopathic, immune-mediated, or other interventions to induce the death of the infected cells, a strategy that has been called ‘shock and kill’ [1].

One drug class being explored to activate HIV-1 transcription is histone deacetylase inhibitors (HDACi) that include vorinostat (SAHA), romidepsin, and panobinostat. These drugs can induce HIV-1 expression without globally activating the immune system [2], and in clinical trials have resulted in increased levels of unspliced cell associated HIV-1 RNA [3, 4] and increased viraemia [5].

However, HIV-1 expression alone may not be sufficient for viral clearance as infected cells may persist despite producing HIV-1 proteins [6]. Therefore an additional ‘kill’ strategy will be required for successful clearance of infected cells. Although CD8+ve cytotoxic T cells (CTL) kill HIV-1-infected cells and are the most likely effectors in ‘shock and kill,’ HDACi may inhibit the CTL response [7]. Additionally, HDACi can also induce HLA class I down-regulation, potentially impairing antigen presentation to CTL. For example, HDACi may induce HIV-1 Nef expression which would subsequently lead to HLA class I down-regulation [8], or HDACi may directly affect HLA class I levels independently of Nef as has been found in several cell lines [9–11]. Unless this can be addressed, these data suggest that the CTL response may not be sufficient for killing cells after HDACi-induced HIV-1 expression. Natural killer (NK) cells also clear infected cells, particularly when HLA class I expression is reduced, and so theoretically may provide an alternative approach to eliminating cells stimulated from latency by HDACi.

Here, we show that the HDACi vorinostat, panobinostat, and romidepsin down-regulate HLA class I levels *ex vivo* independently of HIV-1 Nef to levels sufficient to lead to NK cell degranulation, even without HIV-1 infection. HDACi treatment of infected cells also led to increased NK cell mediated clearance. However, NK cell function was inhibited following treatment with HDACi, indicating that the negative effects of HDACi on NK cells will need to be addressed before NK cells can be used as effective agents of reservoir clearance in clinical trials.

## Results

### HDACi down-regulate HLA class I in primary CD4 T cells independently of Nef

**Fig 1:**
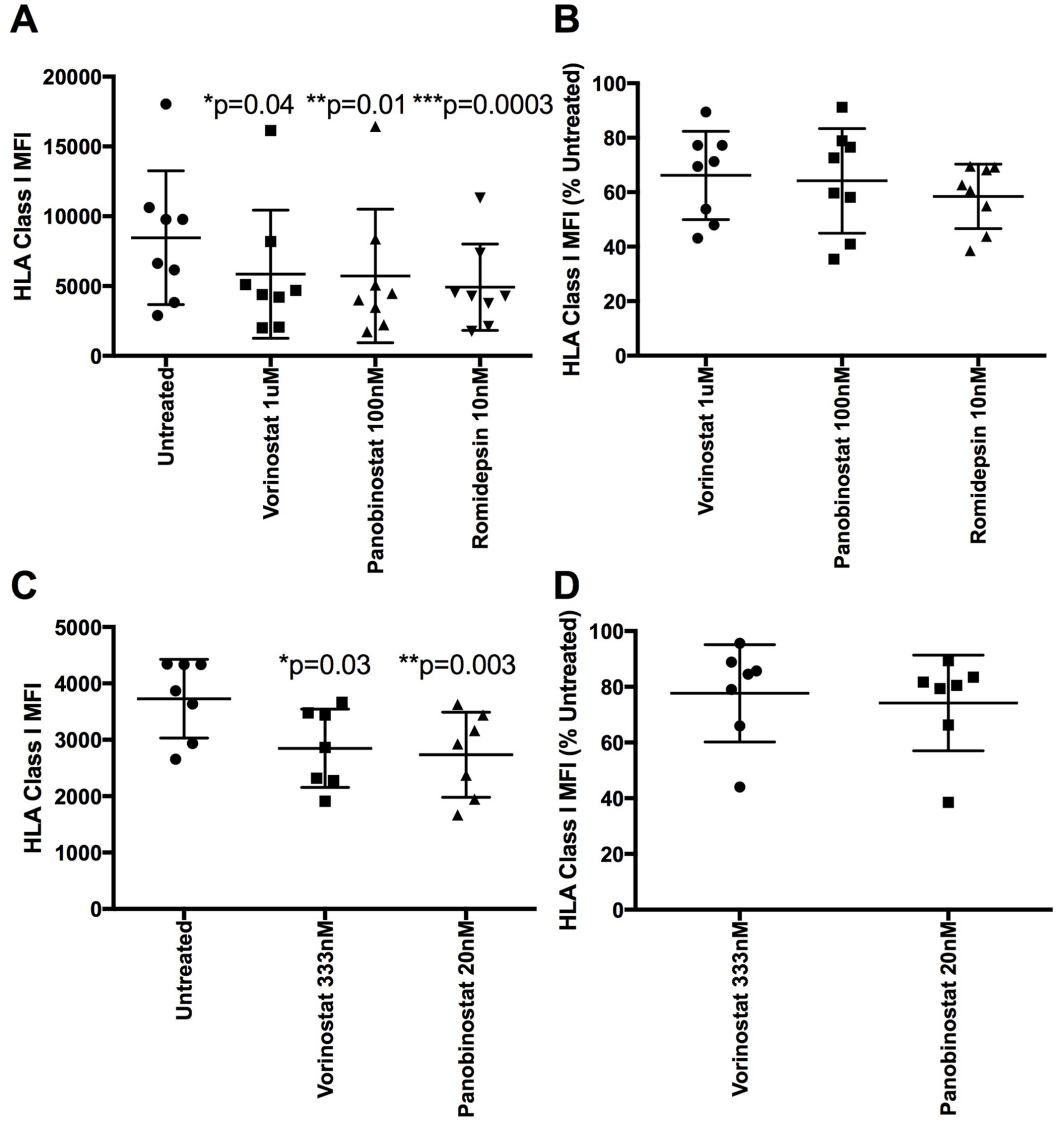
HDACi down-regulate HLA class I in uninfected primary CD4 T cells. A) Unstimulated CD4 T cells from eight HIV negative donors were treated with 1uM vorinostat, 100nm panobinostat or 10nM romidepsin for 24h. Cells were stained for HLA class I. Median fluorescent intensity (MFI) is shown (n = 8). A Friedman test with Dunn’s test for multiple comparisons was performed. B) Data from A was normalized to the MFI of the untreated control and shown as a percentage. C) Culture of primary CD4 T cells from seven new donors was repeated using 333nM vorinostat and 20nM panobinostat (n = 7). A Friedman test with Dunn’s test for multiple comparisons was performed. D) Data from C was normalized to the MFI of the untreated control and shown as a percent.

We first examined the impact of HDACi on HLA class I in uninfected primary CD4 T cells (as cell lines may not accurately reflect *in vivo* effects). We initially cultured uninfected CD4 T cells for 24 hours in the presence of 1μM vorinostat, 100nM panobinostat, or 10nM romidepsin. All three HDACi significantly lowered HLA class I expression in uninfected CD4 T cells compared to untreated controls. The MFI (mean+/- S.D.) for untreated cells was 8464 +/-4797, compared with vorinostat 5851 +/- 4587 (p = 0.04), panobinostat 5724 +/- 4786 (p = 0.01), and romidepsin 4920 +/-3094 (p = 0.0003) (Fig 1A). Converting these data to percentage expression confirmed a substantial reduction in HLA class I expression compared with normal levels (%, S.D.): 66.2+/-15.2% for vorinostat, 64.2 +/- 18% for panobinostat, and 58.5+/-11% for romidepsin (Fig 1B). Using lower, more clinically relevant doses of vorinostat and panobinostat (333nM and 20nM, respectively) [7] also resulted in a significant reduction in HLA class I expression in primary CD4 T cells (vorinostat p = 0.03, panobinostat p = 0.003) (Fig 1C) to approximately 77.7+/- 17.5% and 74.2+/-17.2% of untreated levels respectively (Fig 1D). We verified the results over a range of drug concentrations (S1 Fig) and confirmed the results were not caused by cell toxicity, particularly at the clinically relevant doses where the average viability of each of the HDACi treated cells was >93% compared to untreated controls (S1 Fig). As this effect was demonstrated in uninfected primary cells, the mechanism was HIV-1 Nef-independent. We therefore sought to determine the mechanism behind the lowered HLA class I expression.

### HDACi-induced HLA class I down-regulation is not due to transcriptional blocks or HLA class I internalization

**Fig 2:**
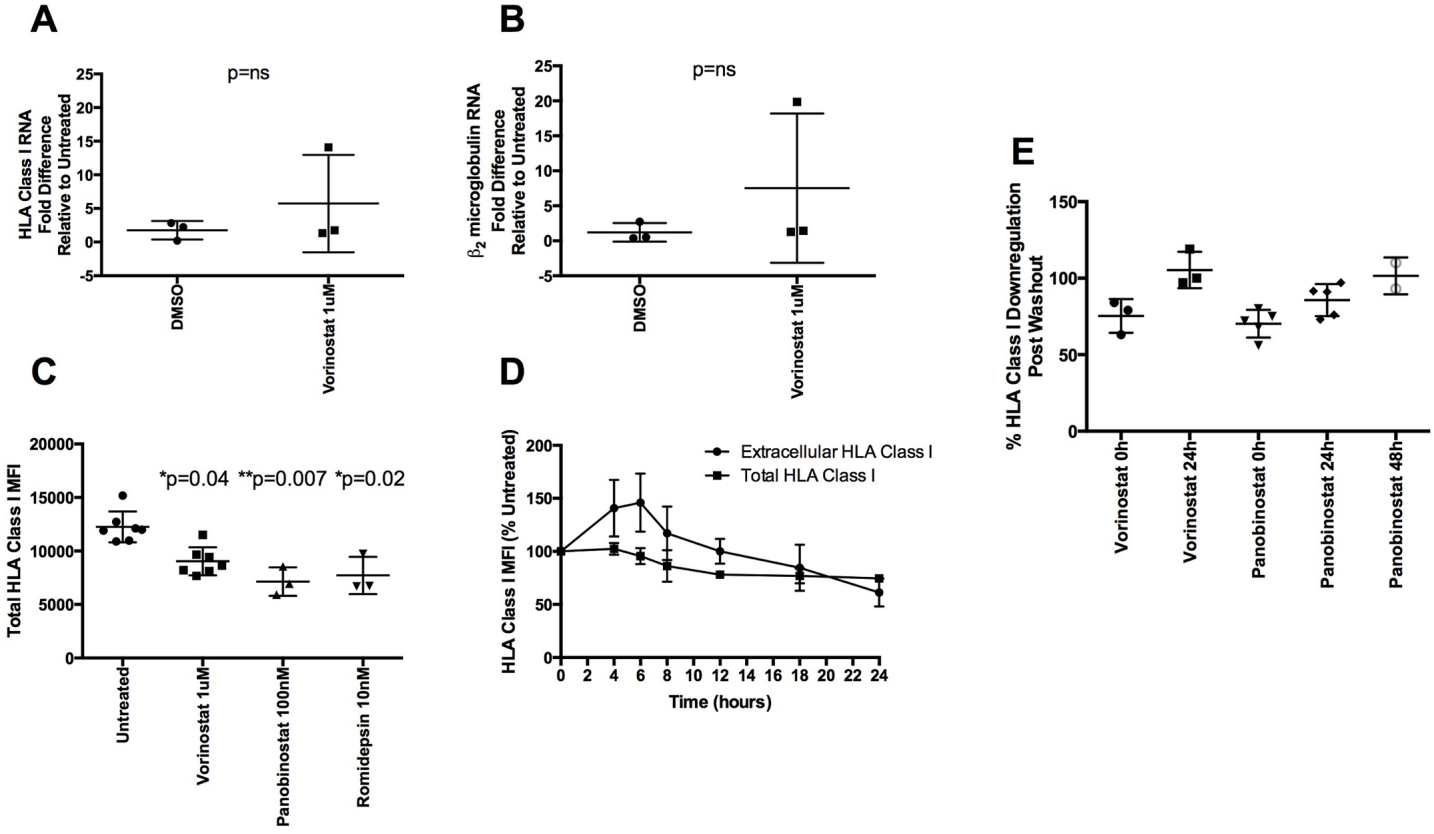
Mechanism of HLA class I down-regulation. (A, B) Fold up-regulation of HLA class I and β_2_ microglobulin RNA, respectively, by qPCR of DMSO and 1uM vorinostat treated samples normalized to untreated controls. All amounts were normalized to 18S copies (n = 3). A Mann-Whitney U test used to compare levels of mRNA expression. C) MFI of total levels (after cell permeabilization) of HLA class I after 24h of culture with HDACi-free media (n = 7), 1uM vorinostat (n = 7), 100nM panobinostat (n = 3) and 10nM romidepsin (n = 3). A Kruskal Wallis test with Dunn’s test for multiple comparisons was performed. D) Kinetics of extracellular and total levels of HLA class I are shown. Extracellular and total values were calculated as a percentage of their respective 0h timepoint MFI (n = 3). E) Cells were treated with 1uM vorinostat or 100nM panobinostat for 24h. Cells were then either washed 3 times or left alone and cultured until HLA class I levels matched untreated controls. Times shown are post wash. Percent down-regulation of HLA class I compared to untreated controls is shown for vorinostat (n = 3) and panobinostat (n = 5).

As HDACi affect transcriptional profiles, we first measured the expression of HLA class I mRNA in HDACi treated uninfected primary cells, under the same culture conditions used above. We found no significant difference in levels of HLA class I mRNA in cells treated with 1μM vorinostat compared to DMSO treated controls (Fig 2A). Similar results were found using panobinostat (S2A Fig). We also measured β_2_ microglobulin RNA levels following HDACi treatment–a protein that is also required for HLA class I cell surface presentation. Similar to HLA class I RNA, we found no significant difference in β_2_ microglobulin levels after treatment with vorinostat or panobinostat (Figs 2B and S2B).

Having demonstrated decreased surface protein expression of HLA class I in the absence of a decrease in HLA Class I or β_2_ microglobulin mRNA levels, we tested whether HDACi led to internalization of HLA class I. To do this, total levels of HLA Class I were assayed following cell permeabilisation to measure both intracellular and surface amounts. All HDACi tested significantly lowered total levels of HLA class I (vorinostat p = 0.04, panobinostat p = 0.007, romidepsin p = 0.02), suggesting that HDACi lead to a global reduction in HLA class I, rather than just expression at the cell surface (Fig 2C).

To understand the relationship between intracellular and extracellular HLA Class I levels following HDACi treatment, we conducted a time-course experiment measuring both over a 24 hour period following 1μM vorinostat treatment. Although total HLA class I levels declined steadily over 24 hours, vorinostat led to a brief increase in extracellular levels after 4 hours of treatment before declining again 12 hours post treatment and continuing to decline thereafter (Fig 2D). This observed rapid effect of HDACi on HLA class I is consistent with the timing of increased cell associated HIV-1 RNA detected in clinical studies [4]. These time-course data indicate that HLA class I was not rapidly internalized after HDACi treatment as this would have led to a decrease in extracellular levels without changing total levels of HLA class I. As the reproducible HDACi reduction of HLA Class I was impacting total protein levels rather than inducing internalization, we tested whether HDACi were causing enhanced proteasomal degradation of HLA class I, using the proteasome inhibitors MG132 or bortezomib with or without HDACi. However, all proteasome inhibitors tested down-regulated HLA class I to a greater degree than the HDACi (S3 Fig), so any HDACi-specific inhibition could not be distinguished.

Finally, we wanted to test the duration of the HDACi effect on HLA class I levels. We treated cells for 24 hours with HDACi and then either left them in culture with drug or washed them three times and cultured them in media alone. Extracellular HLA class I levels were then measured. All vorinostat treated cells had normal levels of HLA class I 24 hours after washing out the drug while panobinostat treated cells took up to 48 hours post washout for normal levels to return (Fig 2E) suggesting a dynamic regulation of HLA class I levels rather than the sustained level of transcription described by others [4].

Having shown HLA class I down-regulation in uninfected CD4 T cells, we turned to CD4 T cells from HIV-1-infected individuals receiving ART.

### HDACi down-regulate HLA class I in CD4 T cells from ART treated patients

**Fig 3:**
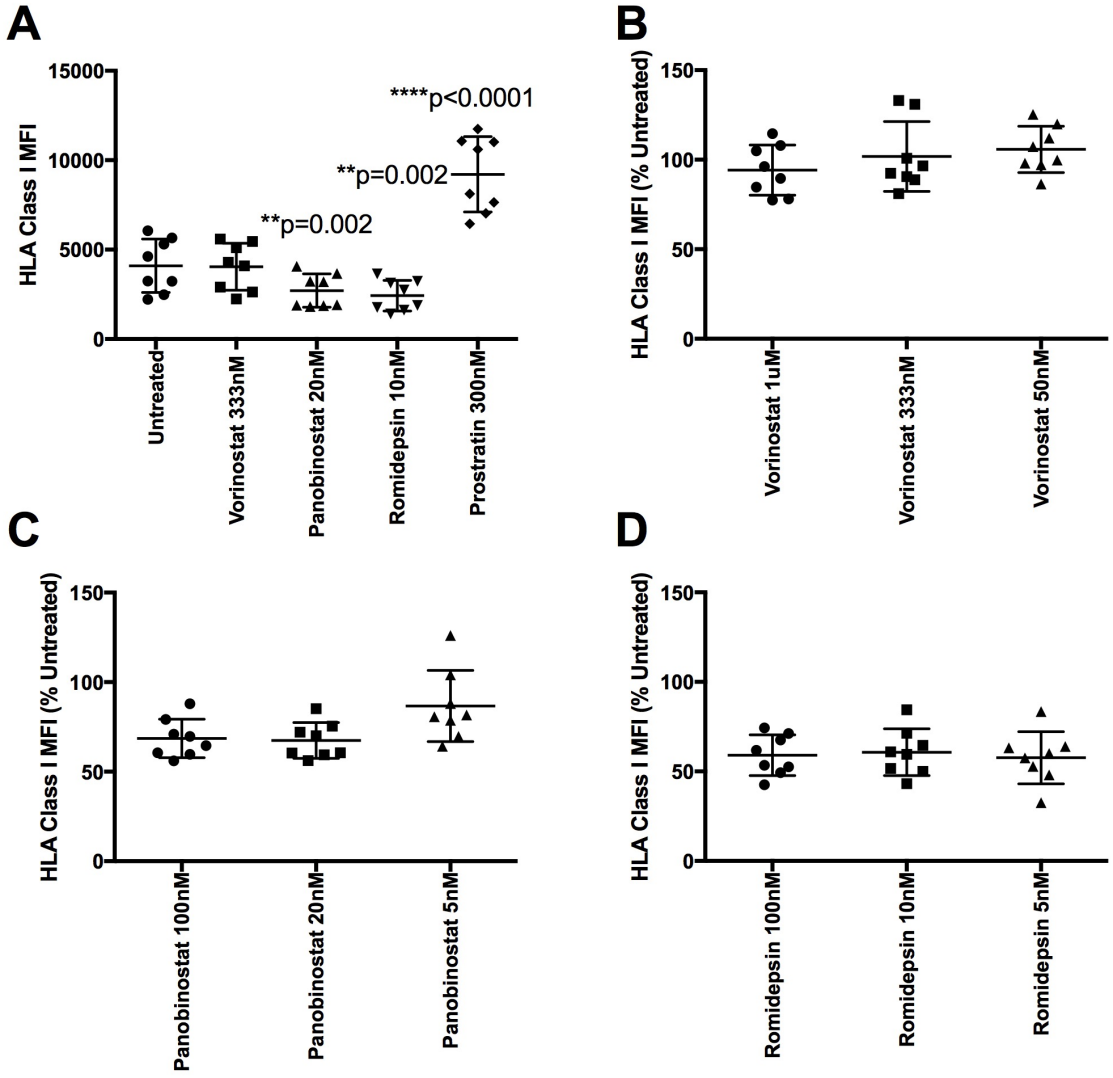
HDACi down-regulate HLA class I expression in patient samples. Fresh CD4 T cells from eight ART treated patients were treated for 24 hours with vorinostat (1uM, 333nm and 50nM), panobinostat (100nM, 20nM, and 5nM), romidepsin (100nM, 10nM, and 5nM) or 300nM prostratin. A) Median fluorescent intensity (MFI) values after treatment with clinically relevant doses are shown. A repeated measures one-way ANOVA with a Greenhouse-Geisser correction with Dunnett’s multiple comparison test was performed. B-D) MFI values reported as a percent reduction compared to untreated levels for vorinostat, panobinostat, and romidepsin dilution series, respectively.

We isolated fresh CD4 T cells from participants (n = 8) in the HEATHER (‘HIV Reservoir Targeting with Early Antiretroviral Therapy’) cohort of individuals treated with ART since primary HIV infection (PHI) (S1 Table). Cells were treated for 24 hours with 3 doses of vorinostat, panobinostat, and romidepsin as well as the PKC activator prostratin (Fig 3). We found that clinically relevant doses of panobinostat and romidepsin significantly reduced HLA class I levels in patient samples (p = 0.002 for both HDACi) while prostratin significantly increased HLA class I expression (p<0.0001). Vorinostat did not significantly reduce HLA class I expression. These results were not the result of cellular toxicity as the HDACi-treated patient samples had similar viability to the HDACi untreated samples (S4 Fig). Of note, the mean percent reduction of HLA class I expression in healthy controls and ART treated patients were similar for both 20nM panobinostat (74.2% vs. 67.4% respectively) and 10nM romidepsin (67.4% vs 60.71% respectively).

### HDACi-induced HLA class I down-regulation does not significantly impact CTL recognition

**Fig 4:**
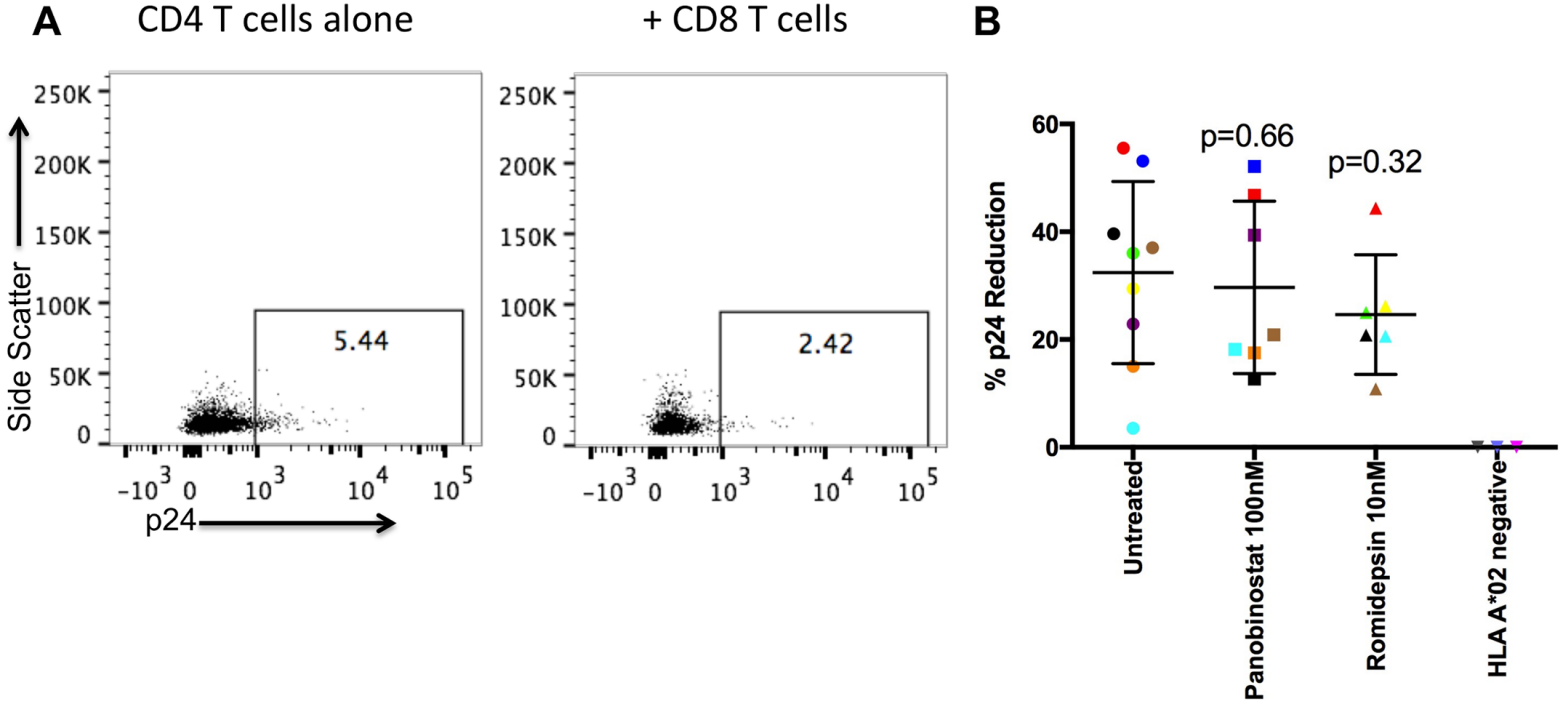
HDACi do not inhibit CD4 T cell susceptibility to CTL killing. HIV-1 LAI infected CD4 T cells with or without HDACi treatment (targets) were cultured overnight with uninfected CD4 T cells with or without HDACi treatment (non-targets) and SL9 transduced CD8 T cells (effectors) at an E:T:NT ratio of 1:1:1. HLA class I A*02 negative donors (n = 3) were used as a control. A) Representative plots of infected CD4 T cells in the absence (left) or presence (right) of CD8 T cells. B) Percent p24 reduction was calculated based on the percentage of p24 positive target cells before and after CD8 T cell co-culture, using 100nM panobinostat treated targets (n = 7) and 10nM romidepsin treated targets (n = 6). Different colors represent different donors. Separate Mann-Whitney U tests were performed.

As HDACi down-regulate HLA class I in CD4 T cells, we tested whether this would impact CTL recognition under optimised conditions. HLA class I A*02 expressing CD4 T cells were infected with HIV-1 LAI, and treated with or without 100nM panobinostat or 10nM romidepsin. We first confirmed HDACi reduced HLA Class I expression in our *in vitro* infected cells (S5 Fig). Then, infected cells were co-cultured with CD8 T cells that were transduced to express a TCR with a high affinity for the HIV-1 Gag peptide SLYNTVATL (SL9), which is expressed on the majority of HLA A*02 infected cells [12]. HIV-1 p24 expression before and after co-culture was assayed as a measure of CTL killing. The percentage of cells expressing p24 was reduced post co-culture consistent with CTL targeting (Fig 4), but with no significant difference between the untreated and the HDACi-treated CD4 T cells, and no p24 reduction when HLA A*02 negative donors were used (Fig 4B). There was no evidence for any significant effect on viability with HDACi treatment on *in vitro* infected CD4 T cells (S4 Fig p>0.1), although there was donor-to-donor variation. (In particular, in two samples of the 100nM panobinostat treated cells there was a substantial reduction in viability (~60% of untreated)). Of note, where there was variation in target viability, this did not impact p24 reduction. Additionally, each killing assay with HDACi had its own control where CTL were not added to control for drug toxicity.

### HDACi treated CD4 T cells induce NK cell degranulation

**Fig 5:**
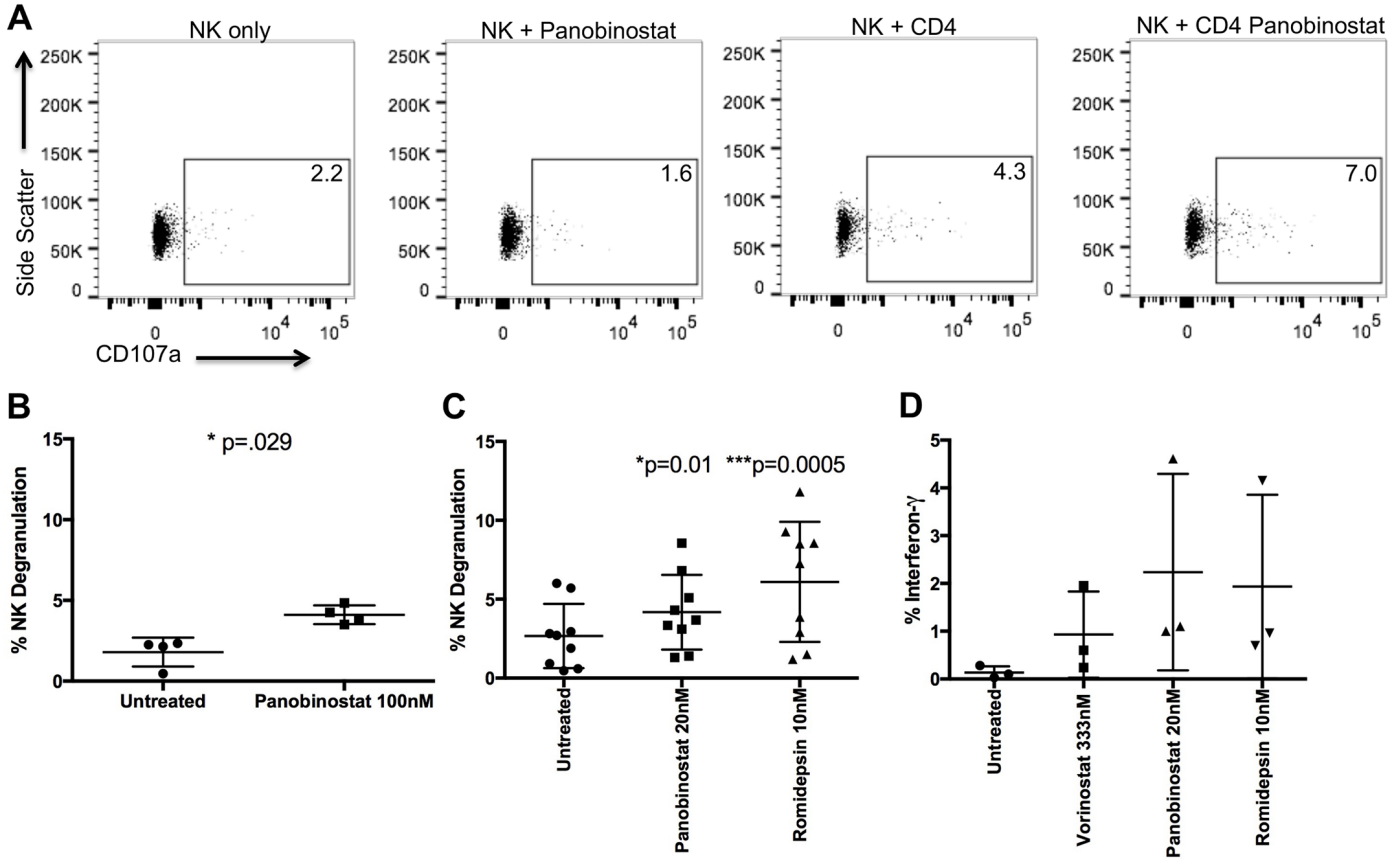
HDACi treatment of CD4 T cells induces NK cell degranulation. Uninfected CD4 T cells were treated or not with HDACi for 24h and then cultured with NK cells for 5h and stained for extracellular CD107a. A) Representative plot showing CD107a expression in NK cells alone, NK cells treated with 100nM panobinostat for 5h, untreated NK cells co-cultured with CD4 T cells at a 1:1 ratio and untreated NK cells co-cultured with 100nM panobinostat treated CD4 T cells at a 1:1 ratio. B) Mean of four experiments. A Mann-Whitney U test was used. C) CD4 T cells were treated with 20nM panobinostat and 10nM romidepsin and co-cultured with NK cells as in A (n = 9). A Friedman’s test with Dunn’s test for multiple comparisons was performed. D) NK cells were co-cultured with untreated CD4 T or CD4 T cells treated with 333nM vorinostat, 20nM panobinostat, or 10nM romidepsin in the presence of 10μg/mL brefeldin A and cells were intracellularly stained with antibodies against IFN-γ.

HLA Class I down-regulation should result in increased NK cell targeting of HDACi treated CD4 T cells. We, therefore, co-cultured primary NK cells at a 1:1 E:T ratio for 5 hours with autologous, uninfected CD4 T cells treated or not with 100nM panobinostat and tested for NK cell degranulation by measuring extracellular CD107a. There was significantly more NK degranulation following co-culture with treated versus untreated targets (p = 0.029; untreated (%, S.D.) 1.8 +/- 0.9 vs panobinostat 4.1% +/- 0.6) (Fig 5A and 5B). This degranulation was not due to an HDACi effect on NK cells as controls without CD4 T cell targets did not show increased CD107a expression (Fig 5A). We tested several E:T ratios to confirm the CD4 T cells were the cause of NK degranulation and compared the level of degranulation to co-cultures using K562 cells, which express no HLA class I, as targets (S6 Fig). NK degranulation decreased as fewer CD4 T cells were added, and co-culture with K562 cells led to more degranulation than with HDACi treated CD4 T cells (S6D Fig), consistent with residual HLA class I expression on CD4 T cells after HDACi treatment. We repeated the co-culture experiments using several doses of vorinostat, panobinostat, and romidepsin (S6A–S6C Fig) including 20nM panobinostat and 10nM romidepsin, which also led to significantly more NK cell degranulation (p = 0.01 panobinostat, p = 0.0005 romidepsin) (Fig 5C). Only the highest vorinostat dose showed a similar increase in degranulation while the clinically relevant doses did not (S6A Fig). In addition to degranulation, NK cells cultured with HDACi treated CD4 T cells showed increased IFN-γ and TNF-α production, compared with almost no detectable cytokine levels (similar to levels of NK cells alone) when cultured with untreated CD4 T cells (Figs 5D and S6E).

### HDACi increase the susceptibility of CD4 T cells to NK killing

**Fig 6:**
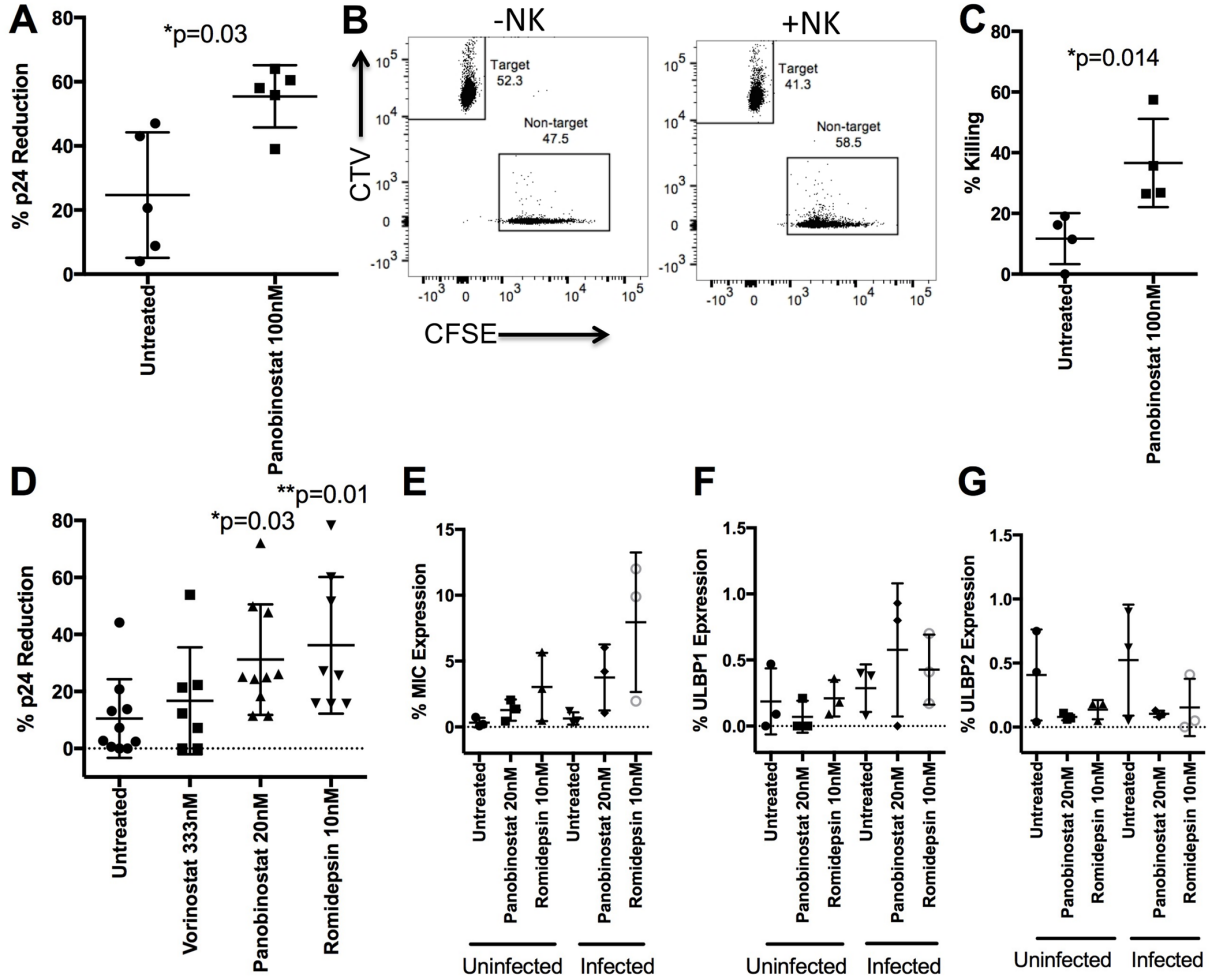
HDACi treatment of CD4 T cells increases NK mediated killing. HIV-1 LAI infected CD4 T cells +/- HDACi (targets) were cultured overnight with uninfected CD4 T cells (non-targets) with or without NK cells (effectors) at an E:T:NT ratio of 10:1:1. A) CD4 T cells were treated with 100nM panobinostat and co-cultured with NK cells overnight. The percent p24 reduction with NK co-culture is shown (n = 5). A Mann-Whitney U test was used. B) A representative FATAL assay with Cell Trace Violet (CTV) stained infected CD4 T cells (targets), CFSE stained uninfected CD4 T cells (non-targets) +/- NK cells. C) Mean of four experiments using the FATAL assay. A Mann-Whitney U test used. D) CD4 T cells were treated with 333nM vorinostat (n = 7), 20nM panobinostat (n = 10) and 10nM romidepsin (n = 8) before culture with NK cells. Percent p24 reduction is shown. A Kruskal-Wallis test with Dunn’s test for multiple comparisons was performed. E-G) Uninfected CD4 T cells and infected cells 48 hours post infection were treated for 24 hours with 20nM panobinostat or 10nM romidepsin. Cells were then stained for levels of E) MICA/B, F) ULBP1, and G) ULBP2 (n = 3).

As HDACi treatment of CD4 T cells led to increased NK degranulation and cytokine production, we tested whether this translated into increased target killing. CD4 T cells infected with HIV-1 LAI were cultured with or without 100nM panobinostat for 24 hours, at 48 hours post infection. We then co-cultured these infected targets overnight with non-target uninfected CD4 T cells (NT) with or without NK effectors at an E:T:NT ratio of 10:1:1 and measured HIV-1 p24 expression (Fig 6A). There was a significantly larger reduction in p24 expression in infected CD4 T cells treated with panobinostat than those without HDACi treatment (p = 0.03, Fig 6A).

As NK cells might inhibit p24 expression without killing infected cells [13], we confirmed that the reduction in p24 reflected actual killing of infected targets by performing a FATAL assay, as previously described [14] (Fig 6B and 6C). Briefly, infected targets were stained with a Cell Trace Violet (CTV) dye while non-target cells were stained with a Cell Trace CFSE dye. The ratio of targets to non-targets with and without NK cells was calculated, and the change in this ratio after NK co-culture was converted to percentage killing. NK cells preferentially targeted and killed infected cells, evidenced by a lower target to non-target ratio after NK cell co-culture (Fig 6B and 6C). A significantly larger fraction of infected cells were cleared following treatment with 100nM and 20nM panobinostat (p = 0.014, Fig 6C and p = 0.03, Fig 6D, respectively) and 10nM romidepsin (p = 0.01 Fig 6D), compared to untreated controls, but not with vorinostat. Increased target killing was unlikely to be due to increased HIV-1 production as there was no evidence for higher levels of p24 in HDACi treated cells before NK co-culture despite higher levels of HIV-1 RNA (S7 Fig), consistent with some prior work showing HDACi alone did not induce HIV-1 expression in *ex vivo* stimulated patient samples [15]. We performed the same experiments with a range of HDACi concentrations with similar results (S8 Fig).

Expression of NK activating ligands MIC A/B, ULBP1 and ULBP2 on CD4 T cells was measured to determine potential mechanisms of the preferential killing of infected cells. CD4 T cells were isolated from healthy donors and half were infected, as above. After 48h of infection both uninfected and infected cells were stimulated with 20nM panobinostat or 10nM romidepsin using untreated cells as a negative control. Cells were stained for MIC A/B, ULBP1 and ULBP2, all ligands for the activating NKG2D receptor found on NK cells. HDACi treatment led to increased MIC A/B expression on uninfected cells with a greater increase with HIV-1 infection (Fig 6E), although there was no difference in ULBP1 or 2 levels with or without HDACi treatment (Fig 6F and 6G respectively) suggesting MIC A/B levels could explain the preferential killing of infected cells through the NKG2D pathway.

### HDACi inhibit NK Cell killing

**Fig 7:**
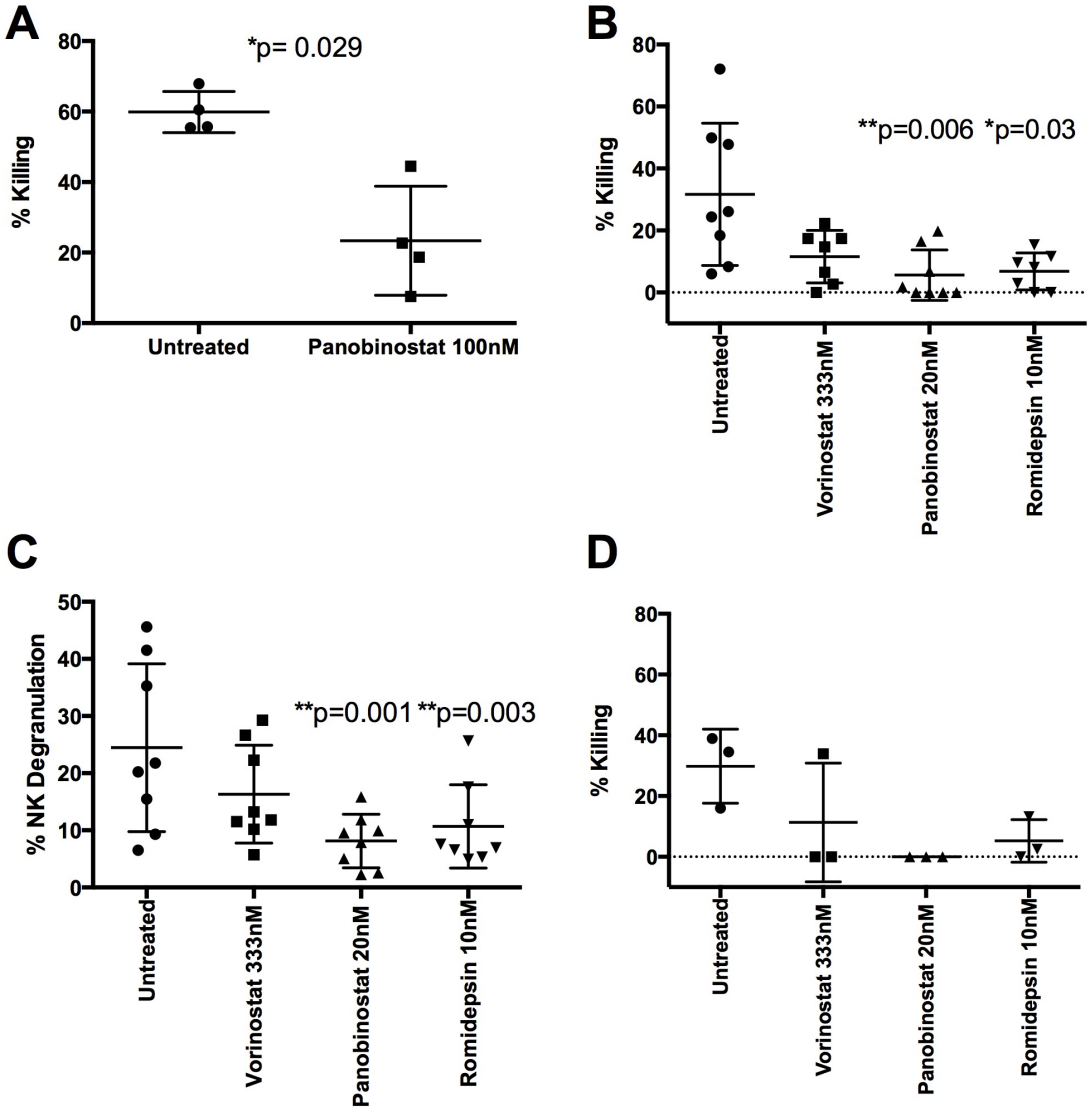
HDACi decrease NK function. A) NK cells treated with or without HDACi (as PBMC) for 24h (effectors) were co-cultured or not with HIV-1 LAI-infected 100nM panobinostat treated CD4 T cells (targets) and uninfected CD4 T cells (non-targets) overnight at an E:T:NT ratio of 10:1:1 (n = 4). Percent killing based on p24 reduction is shown and a Mann-Whitney U test was performed. B) NK cells were treated with 333nM vorinostat (n = 7), 20nM panobinostat (n = 8) and 10nM romidepsin (n = 7). Targets were infected CD4 T cells treated with 20nM panobinostat. A Kruskal-Wallis test with Dunn’s test for multiple comparisons was performed. C) NK cells treated with or without 333nM vorinostat, 20nM panobinostat, or 10nM romidepsin were cultured for 5h with K562 cells at a 1:1 ratio after which extracellular CD107a was measured (n = 8). A Friedman test with Dunn’s multiple comparison test was performed. D) A FATAL assay was performed using NK cells treated with or without 333nM vorinostat, 20nM panobinostat, or 10nM romidepsin (effectors) which were co-cultured with Cell Trace Violet labeled K562 cells (targets) and CFSE labeled THP1 cells (non-targets) at a 1:1:1 ratio overnight. The ratio of targets:non-targets with and without NK cells was then used to determine percent killing (n = 3).

The inhibitory effects of HDACi on CTL highlight the importance of examining the effects of HDACi not only on potential targets but also on effectors [7]. We therefore examined the direct effect of HDACi on NK function, by testing the ability of 100nM panobinostat treated NK cells to kill HDACi treated infected targets. HDACi-treated NK cells killed significantly fewer cells than untreated NK cells [untreated (% killed, S.D.) 60.0+/- 5.8 vs panobinostat 23.4 +/- 15.5; p = 0.029] (Fig 7A). 333nM vorinostat, 20nM panobinostat and 10nM romidepsin also reduced NK killing, although this was only significant for the latter two (p = 0.006, p = 0.03 respectively) (Fig 7B). We performed the same experiments using several dilutions of each HDACi with evidence for a dose-related response (S9 Fig). These results were likely not due to cytotoxic effects of HDACi as only the highest, non-physiological doses impacted cell viability (S9 Fig).

To determine the mechanism behind the reduced killing we first measured the ability of HDACi treated NK cells to degranulate in response to the HLA class I negative cell line K562. We co-cultured NK cells treated or not with 333nM vorinostat, 20nM panobinostat and 10nM romidepsin with K562 cells at a 1:1 ratio for five hours and measured extracellular CD107a expression. Both panobinostat and romidepsin treatment resulted in significantly less degranulation than untreated controls (Fig 7C p = 0.001, p = 0.003 respectively), while vorinostat treated cells did not (Fig 7C). Experiments were repeated with a wide range of HDACi doses and supported a dose-related response (S10 Fig). We tested whether reduced degranulation resulted in less killing of K562 cells, using the FATAL assay with K562 as targets, THP1 cells as non-targets and NK cells treated with or without 333nM vorinostat, 20nM panobinostat, or 10nM romidepsin as effectors. HDACi that resulted in less degranulation also resulted in less killing of K562 cells (Fig 7D).

To understand if HDACi might phenotypically alter NK cells, making them less effective killers, we began by examining CD16 which is associated with cytotoxicity [16]. 100nM panobinostat significantly reduced CD16 expression (p = 0.01) while lower doses of panobinostat and romidepsin did not (S11A Fig). We also examined expression of the NK activating receptors NKp46 and NKG2D. Similar to CD16 expression, only 100nM panobinostat significantly reduced NKp46 expression (p = 0.0002, S11B Fig). However, both doses of panobinostat significantly reduced NKG2D expression (p = 0.001, p = 0.04; S11C Fig) while romidepsin did not, suggesting that different HDACi can have different effects on NK phenotype while still inhibiting NK function.

### Variable outcomes following HDACi treatment of both infected CD4 T cells and NK cells

**Fig 8:**
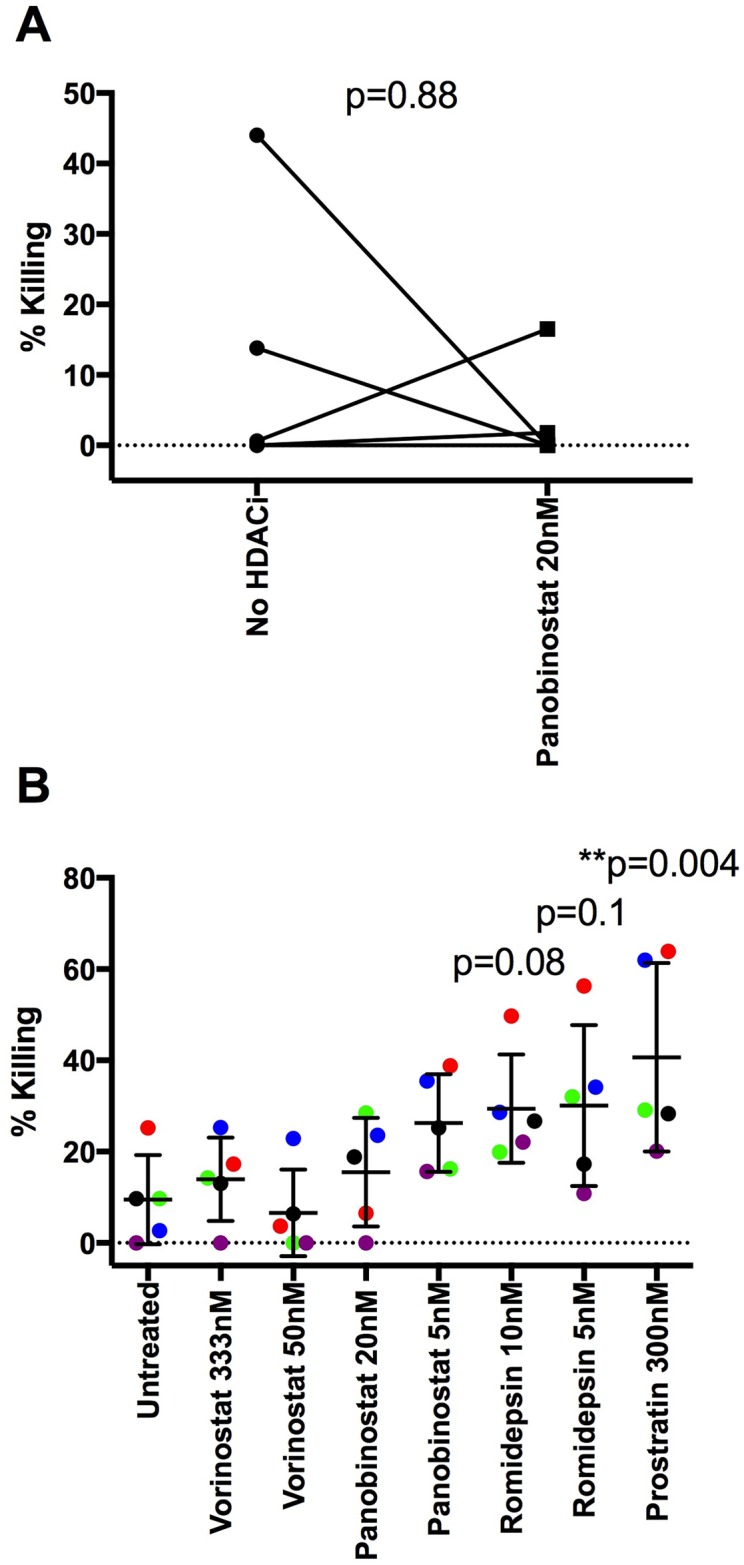
Effects of treating both CD4 T cells and NK cells with HDACi depends on the chosen HDACi. A) An NK co-culture assay was performed overnight at a 10:1:1 E:T:NT ratio with either infected, untreated CD4 T (targets) and untreated NK cells (effectors) or with 20nM panobinostat treated targets and HDACi treated effectors. Intracellular p24 was measured and compared between the samples with and without NK cells. The difference in p24 expression was then converted to percent killing as described. A Wilcoxon matched-pairs signed rank test was performed (n = 5). In B, a similar co-culture assay was performed using 2 doses of vorinostat (333nM and 50nM), 2 doses of panobinostat (20nM and 5nM), 2 doses of romidepsin (10nM and 5nM) and 300nM prostratin. A Friedman test with Dunn’s multiple comparison test was performed (n = 5). Different colors represent different donors.

As HDACi directly inhibited NK cell function, but also rendered HIV-1-infected CD4 T cells more susceptible to killing, we wanted to determine the overall effect when both were treated. For five individual donors, we treated either both or neither CD4 T and NK cells with 20nM panobinostat. Overall, there was no significant difference in killing between the two, suggesting the negative effects of treating NK cells with HDACi cancelled out any benefits of treating targets cells with panobinostat (p = 0.95 Fig 8A). However, there was donor-to-donor variation: two of five donors showed higher levels of killing, two showed reduced killing and one donor showed no difference. To determine if this result was drug or dose dependent, the experiment was repeated with a further five donors using two doses of vorinostat (333nM and 50nM), panobinostat (20nM and 5nM), and romidepsin (10nM and 5nM) as well as 300nM prostratin. Prostratin significantly enhanced NK killing (p = 0.005) despite significant effects on cell viability (S9G Fig), while there was weak evidence for both 10nM and 5nM doses of romidepsin enhancing killing (p = 0.08 and p = 0.1 respectively) (Fig 8B). Interestingly, for both vorinostat and panobinostat there was again evidence for donor-donor variation while romidepsin showed no such variation (Fig 8B). Overall, these data suggest that different HDACi have varied effects on NK mediated killing when both effectors and targets are treated, but that romidepsin may allow more effective NK-directed clearance of the HIV-1 reservoir.

## Discussion

In this study we examined the effects of HDACi on HLA class I expression and the impact on CTL and NK cell targeting. We found that all tested HDACi (vorinostat, panobinostat, and romidepsin) down-regulated HLA class I expression in a Nef-independent manner. Although HLA class I down-regulation did not significantly impact CTL recognition, it did lead to increased NK degranulation upon co-culture and significantly increased NK cell mediated killing of targets. However, when NK cells were treated with HDACi their killing capacity was reduced. Overall, our results provide insights into the use and pitfalls of NK cells as potential effectors in HDACi based ‘kick and kill’ approaches.

It was surprising that all HDACi tested down-regulated HLA class I as previous studies have found HDACi can up-regulate HLA class I and II molecules on tumour cells and cell lines [9, 17]. However, these studies were not performed using healthy CD4 T cells, and it is possible that HDACi have different effects depending on cell type and health. While HLA class I down-regulation was HIV-1 independent, it is possible that this could lead to preferential targeting of HIV-1 infected cells as NK cell targeting is dictated by a balance of activating and inhibitory signals [18], which may themselves be impacted by HIV-1. Our data showing enhanced MIC A/B expression in infected cells treated with HDACi compared to uninfected HDACi treated cells supports this possibility. We saw a similar reduction in HLA class I in cells from patients on ART treated *ex vivo* with panobinostat and romidepsin. Importantly, the transitory nature of the down-regulation may minimize the potential negative consequences of lower levels of HLA class I in patients.

HLA class I down-regulation led us to examine the effects of HDACi on CTL and NK targeting of infected cells. Prior work showed HDACi limited CTL function *in vitro* upon treatment of CD8 T cells with HDACi [7]. Here, we tested whether HLA class I down-regulation upon HDACi treatment of infected targets might also affect CTL recognition. We found no significant difference in CTL recognition of HDACi treated targets suggesting lower HLA class I levels might not affect CTL clearance to a substantial degree. However, our studies utilized a high affinity SL9 TCR [12] that might not be as impacted by lower levels of HLA class I expression as other TCRs.

As the effects of HDACi on both targets and effectors will impact clearance of HIV-1 infected cells, we examined how HDACi affected both CD4 T cells and NK cells, respectively. Interestingly, HDACi treatment on these two cell types had opposite effects on HIV-1 clearance. CD4 T cells (targets) treated with HDACi were more likely to be cleared than untreated cells. Increased clearance could be due to a combination of factors including lower HLA class I levels and higher levels of NK activating ligands such as MIC A/B. Previous data have shown increased levels of soluble MIC A during HIV-1 infection [19] as well as reduced levels of MIC A on the surface of CD4 T cells in HIV-1 infected patients [20, 21] possibly due to MIC A/B shedding by HIV-1 infected cells [19, 22]. Higher MIC A/B levels caused by HDACi thus might allow killing through MIC mediated pathways. While work has shown the importance of ULBP1 and ULBP2 in NK mediated killing of HIV-1 infected cells [21] we found no significant difference in their expression after HDACi treatment. HDACi treatment of NK cells (the effectors), on the other hand, reduced HIV-1 clearance. This might be due to reduced degranulation or changes to NK phenotype such NKp46, NKG2D and CD16 expression.

We found that different HDACi had varying effects on NK cell phenotype. Both high and low doses of panobinostat reduced NKG2D expression on NK cells, but romidepsin did not. This may be due to the fact that panobinostat is a pan-HDAC inhibitor while romidepsin potently inhibits HDAC 1 and HDAC 2. Despite this differential effect on NK phenotype, both romidepsin and panobinostat treatment of NK cells reduced HIV-1 clearance. This suggests that reduced levels of degranulation may have played a larger role than NKG2D levels in reducing NK mediated killing.

As HDACi impact effectors and targets differently, it was important to know whether treating both would lead to increased or decreased killing. Treating both had variable effects depending on the HDACi used—vorinostat and panobinostat showed donor-donor variation but romidepsin trended towards increased clearance of infected cells with all five donors showing increased NK mediated killing. Previous studies using tumour cells as targets also found treating NK cells with HDACi may decrease their function [23]. However, prior work showed that treating both cancer cells and NK cells with HDACi still led to enhanced tumor cell killing [24, 25]. It is therefore important to examine this balancing act in the appropriate system, due to inherent differences in cancer and HIV-1-infected cells.

All of our experiments were performed *in vitro* or *ex vivo*. The effects of HDACi on NK or CTL targeting/function *in vivo* are still unknown, although analyses of clinical trials currently underway (e.g the RIVER study [26]) will shed light on this. While the limited dosing schedule and half-life of the HDACi *in vivo* may limit effects on the immune system, the short duration of HLA class I down-regulation after removal of HDACi may make it difficult to take advantage of the enhanced susceptibility of HDACi treated CD4 T cells to NK clearance.

In summary, our experiments reaffirm the importance of studying the effects of latency reversing agents (LRA) on multiple components of the immune response including targets and multiple effectors in a complex system where several cell types interact. Overall, in the search for an effective method to target the HIV-1 reservoir, our data suggest that HDACi alone will not be effective within a ‘kick and kill’ strategy, but that other co-administered agents are likely to be required to enhance efficacy or negate any inhibitory effects.

## Materials and Methods

### Ethics statement

Primary human CD4 T cells used in this study were isolated from leukocyte cones obtained through anonymous donation to NHS Blood and Transplant (UK) after informed, written consent and approval by NHS Blood and Transplant and the National Research Ethics Service Oxfordshire Research Ethics Committee.

The HEATHER (‘HIV Reservoir targeting with Early Antiretroviral Therapy’) study was approved by the West Midlands—South Birmingham Research Ethics Committee reference 14/WM/1104. Ethical approvals include use of samples for the studies described. All samples were analysed anonymously.

### Cell culture

CD4 T cells were negatively selected from PBMC using the EasySEP human CD4 T cell enrichment kit (StemCell Technologies) per the manufacturer’s protocol. Cells were then cultured in RPMI containing 10%FCS, penicillin/streptomycin and L-glutamine (R10). HDACi treated cells were cultured in 1μM or 333nM vorinostat (Sigma), 100nM or 20nM panobinostat (Cambridge Bioscience), or 10nM romidepsin (Abcam).

### Drug doses

For vorinostat (Sigma), we chose a range of 50nM– 1 μM. The high dose of 1μM was chosen as it was used in *in vitro* latency experiments and the peak serum concentration of vorinostat *in vivo* was reported to be 1.2μM; however as the free concentration of vorinostat was only 360nM [27], we used 333nM as our physiologically relevant dose. For panobinostat (Cambridge Bioscience), we chose a range from 5-100nM. The mean clinical C_max_ of panobinostat at the 20mg p.o. dosing of the CLEAR trial is 40nM [28]and the steady-state plasma concentration is 15-22nM [7] so we chose 20nM as our physiological dose. For romidepsin (Abcam), we chose a range of 5-100nM to match our panobinostat range. The peak serum concentration for clinical doses is 698nM with free drug concentration being 56nM [27]. We thus chose 10nM, which incorporated our cell line toxicity studies. We used 300nM prostratin based on other *ex vivo* studies [29].

### HIV-1 infection

MT4 cells were transfected with a HIV-1 LAI encoding plasmid via electroporation. Viral supernatant was then harvested 9 days post transfection. Isolated CD4 T cells were spinoculated with this LAI supernatant (100μL per 1e6 cells, MOI 1.8) for 2 hours at 1200xg as in [30]. Cells were then washed twice with R10, resuspended and cultured in R10 with 1.25μM saquinavir. If infected cells were treated with HDACi, drugs were added 48 hours post infection and left in culture for 24 hours.

### HLA class I staining

CD4 T cells treated or not with HDACi were stained first with the LIVE/DEAD fixable near-IR dead cell stain kit (Life Technologies) as per the manufacturer’s protocol along with HLA class I staining using a HLA-ABC clone w6/32 APC antibody (eBioscience). For intracellular staining (total HLA class I), cells were stained with live/dead stain as above and then fixed with 2% paraformaldehyde for 30 minutes. Cells were then washed and then simultaneously permeabilized with 0.05% saponin and stained with HLA-ABC APC for 40 minutes. For the time-course experiment, untreated and HDACi treated cells were stained for extracellular and total HLA class I at t = 0, 4, 6, 8, 12, 18, and 24 hours post HDACi treatment.

### RNA expression

CD4 T cells were cultured in R10, treated with DMSO or treated with HDACi as above. RNA was isolated using the RNeasy Mini Kit (Qiagen). HLA class I RNA was measured using previously described primers 5’-CCTACGACGGCAAGGATTAC-3’ and 5’-TGCCAGGTCAGTGTGATCTC-3’ [31]. B_2_ microglobulin was measured using the forward primer 5’-TCAATGTCGGATGGATGAAA and the reverse primer 5’-GTGCTCGCGCTACTCTCTCT [32]. Expression levels were then normalized to 18s rRNA levels measured using the previously described primers 5’TCGAGGCCCTGTAATTGGAA-3’ and 5’GAGTCCTGCGTCGAGAGAGC [33]. All qPCR reactions were performed with a Roche Lightcycler 480 using the Lightcycler 480 SYBR Green I Master Mix (Roche) using the following program: 1 cycle of 95°C for 10 minutes followed by 45 cycles of 95°C for 10 seconds, 55°C for 25 seconds, and 72°C for 30 seconds.

### CTL assay

HLA Class I A*02 positive CD4 T cells were infected and treated or not with 100nM panobinostat and 10nM romidepsin as above. HLA A*02 negative donors were used as a control. CD8 T cells transduced with TCRs recognizing the HIV-1 SL9 gag peptide were supplied by Adaptimmune. Infected CD4 T cells were stained using the Cell Trace Violet Cell Proliferation Kit (Life Technologies) as per the manufacturer’s protocol. Non-target uninfected CD4 T cells were stained using the Cell Trace CFSE Cell Proliferation Kit (Life Technologies) per the manufacturer’s protocol. CD8 T cells were then co-cultured overnight with targets and non-targets at a E:T:NT ratio of 1:1:1. To measure p24, cells were fixed and permeabilized (as above) and stained using the KC57 PE antibody (Beckman Coulter). p24 expression was then measured in target cells.

### NK cell isolation and HDACi treatment

PBMC were cultured for 24 hours in R10 with or without HDACi. NK cells were then negatively selected using the EasySep Human NK Cell Enrichment Kit (StemCell Technologies) as per the manufacturer’s protocol.

### NK degranulation assay

Uninfected CD4 T cells were isolated as above and treated or not with HDACi at the indicated doses. Meanwhile NK cells were isolated as above. NK and CD4 T cells were cultured at a 1:1, 1:0.2, or 1:0.01 ratio for 5 hours at 37°C in the presence of an anti-CD107a PE-Cy7 antibody (Biolegend). Experiments using K562 cells were performed at a 1:1 ratio for 5 hours as with CD4 T cells. For both CD4 T and K562 experiments, cells were then washed and stained with a panel of: LIVE/DEAD fixable near-IR dead cell stain kit (Life Technologies), anti-CD3 eflour450 (eBioscience), anti-CD56 APC (Miltenyi), anti-CD16 FITC (Biolegend), anti-CD14 APC-Cy7 (Biolegend), anti-CD19 APC Cy7 (Biolegend). NK cells were gated on APC Cy7 negative cells (live, CD14-, CD19-), CD3 negative, CD56 positive cells.

### NK cytokine assay

Uninfected CD4 T cells were isolated as above and treated or not with 333nM vorinostat, 20nM panobinostat or 10nM romidepsin. Meanwhile NK cells were isolated as above. NK and CD4 T cells were cultured at a 1:1 ratio for 5 hours at 37°C. After the first hour of incubation 10μM brefeldin A (Sigma Aldrich) was added to all cells. After the 5 hour incubation cells were extracellularly stained for CD3 and CD56 as above and intracellularly stained with IFN-γ PE-Cy7 and TNF-α PerCP-Cy5.5.

### NK cell p24 reduction and FATAL assay

CD4 T cells were isolated as above. Half of the cells (targets) were infected with HIV-1 as above. The other half was cultured in R10 (non-targets). 48h post infection, infected cells were treated or not with 100nM panobinostat, 20nM panobinostat, or 10nM romidepsin for 24h. Meanwhile NK cells were isolated as above. 72h post infection targets were stained using the Cell Trace Violet Cell Proliferation Kit (Life Technologies) as per the manufacturer’s protocol. Non-targets were stained using the Cell Trace CFSE Cell Proliferation Kit (Life Technologies) per the manufacturer’s protocol. NK cells were then co-cultured overnight with targets and non-targets at a E:T:NT ratio of 10:1:1. In control wells NK cells were not added. After co-culture, cells were stained with LIVE/DEAD fixable near-IR dead cell stain kit (Life Technologies). p24 was measured as above. For the FATAL assay, live cells were gated for targets and non-targets. The number of non-targets:targets (using cell counts from FlowJo) in the wells without NK cells was converted to a NT:T ratio. This ratio was then used to predict the number of targets expected in the wells with NK cells based on their non-target number. The difference between the actual and expected number of targets was then converted into a percent targets killed (% cytotoxicity). For FATAL assays using K562 cells the experiments were performed as above with K562 cells as the targets and THP1 cells as the non-targets and were done using a 1:1:1: E:T:NT ratio overnight.

### MIC and ULBP staining

CD4 T cells were isolated as above. One fraction of cells was infected HIV as above for 48 hours in the presence of saquinavir while the second fraction of cells was left uninfected in culture. After 48 hours, both uninfected and infected cells were treated with or without 20nM panobinostat or 10nM romidepsin after which they were stained with one of two antibody cocktails: 1) LIVE/DEAD fixable near-IR dead stain (Life Technologies)and MIC A/B PE (BD Biosciences) 2) LIVE/DEAD ULBP1 PE (R&D Systems) and ULBP2 FITC (Biorbyt). Antibody function was confirmed in Jurkat cells.

### HDACi and NK phenotyping

PBMC were cultured for 24h in the absence or presence of 100nM panobinostat, 20nM panobinostat, or 10nM romidepsin. After culture, wells were stained with a panel of: LIVE/DEAD fixable near-IR dead cell stain kit (Life Technologies), anti-CD3 eflour450 (eBioscience), anti-CD16 APC (Cambridge Bioscience), anti-CD56 FITC (Cambridge Bioscience), anti-CD14 APC-Cy7 (Biolegend), anti-CD19 APC Cy7 (Biolegend), NKG2D PECy7 (Cambridge Bioscience), and CD335 (NKp46) PE (Miltenyi Biotec). NK cells were gated on APC Cy7 negative cells (live, CD14-, CD19), CD3 negative, CD56 positive cells.

### Statistics

All statistical tests were performed using GraphPad Prism 6 (GraphPad Sofware, Inc.).

## Supporting Information

S1 Fig Dilution series of HLA class I and viability of HDACi treated uninfected cells. Uninfected CD4 T cells were treated for 24 hours with the indicated dose of HDACi. The MFI of HLA class I as a percent of untreated controls is shown for a range of doses of vorinostat (A), panobinostat (C), and romidepsin (E). The viability of these cells is shown in B, D, and F respectively (n = 5)
(TIF)

S2 Fig Panobinostat effect on HLA class I and β_2_-microglobulin RNA. Fold upregulation of HLA class I (A) and β_2_-microglobulin RNA (B) by qPCR of DMSO and 100nM panobinostat treated samples normalized to untreated controls is shown. All amounts were normalized to 18S copies (n = 2).
(TIF)

S3 Fig Proteasome inhibitors reduce HLA class I levels. Healthy primary CD4 T cells were cultured in media (untreated) or treated with 1μM vorinostat, 10μM MG132, 1μM MG132, 1μM bortezomib, or 100nM bortezomib. The MFI of HLA class I levels is shown (n = 3 vorinostat, MG132; n = 2 bortezomib)
(TIF)

S4 Fig Viability of infected CD4 T cells treated with HDACi. The viability of CD4 T cells from HIV infected patients treated with doses of vorinostat, panobinostat, romidepsin and prostratin is shown in A-D respectively (n = 5). The viability of *in vitro* infected cells treated with the same drug doses are shown in E-G (n = 3 vorinostat, prostratin; n = 6 panobinostat; n = 5 romidepsin).
(TIF)

S5 Fig HDACi down-regulate HLA Class I in *in vitro* infected CD4 T cells. CD4 T cells were spinoculated with HIV-1 LAI for 48 hours and then treated with 100nM panobinostat or 10nM romidepin for 24 hours. HLA Class I levels were then measured and reported as a percent of untreated controls (n = 4).
(TIFF)

S6 Fig NK degranulation at different HDACi doses and E:T ratios and TNF-α production upon co-culture. CD4 T cells treated with several doses of vorinostat, panobinostat and romidepsin were co-cultured with NK cells at a 1:1 ratio for 5 hours and CD107a expression was measured in A-C respectively (n = 4). In D, either CD4 T cells treated with or without 100nM panobinostat or untreated K562 cells were co-cultured with NK cells at a 1:1, 1:0.2, or 1:0.1 E:T ratio (n = 3). E) TNF-α production was measured in NK cells co-cultured for 5 hours with cells treated with or without 333nM vorinostat, 20nM panobinostat, or 10nM romidepsin (n = 3).
(TIF)

S7 Fig p24 and RNA levels in HDACi treated cells. CD4 T cells were infected with LAI for 48 hours after which they were either left in media or treated for 24 hours with 1μM vorinostat or 100nM panobinostat. Intracellular p24 levels (A) and cell- associated unspliced HIV-RNA (B) were measured 72 hours post infection (n = 4). C) Cells were infected as above and treated with 333nM vorinostat, 20nM panobinostat, and 10nM romidepsin for 24 hours. Intracellular p24 levels are shown (n = 5)
(TIF)

S8 Fig HDACi increase CD4 T cell susceptibility to NK mediated killing at several drug doses. *In vitro* infected CD4 T cells were treated for 24h with several doses of vorinostat, panobinostat, and romidepsin in A-C respectively and a killing assay based on p24 reduction was performed as in Fig 6 (n = 3).
(TIF)

S9 Fig Effects of various doses of HDACi treatment of NK cells on NK mediated killing and NK cell viability. NK cells treated with or without several doses of vorinostat, panobinostat, and romidepsin were co-cultured with infected CD4 T cells and a killing assay based on p24 reduction was performed as described (A, C, and E respectively, n = 3). Viability of the NK cells was measured in B, D, and F for the same HDACi doses (n = 5). In G, viability of NK cells treated with 300nM prostratin was measured (n = 5).
(TIF)

S10 Fig Effects of several doses of HDACi on the degranulation of NK cells co-cultured with K562 cells. NK cells were treated with various of doses of vorinostat, panobinostat, and romidepsin (A-C respectively) and co-cultured or not with K562 cells for 5 hours at a 1:1 ratio. CD107a expression was measured (n = 5).
(TIF)

S11 Fig Effects of HDACi on NK phenotype. PBMC were cultured with or without 100nM panobinostat, 20nM panobinostat, or 10nM romidepsin for 24h. NK cells were gated using live/dead and lineage markers. The percentage of CD16hi (A) NKp46 (B) and NKG2D (C) expressing cells is shown (n = 6). A Friedman test with Dunn’s test for multiple comparisons was performed.
(TIF)

S1 Table Patient characteristics. Patient characteristics of the eight HEATHER patients used for HLA Class I measurements.
(PDF)
