# Supplementary figures and images for: Histone Deacetylase Inhibitors Enhance CD4 T Cell Susceptibility to NK Cell Killing but Reduce NK Cell Function
Source: PLoS Pathog. 2016 Aug 16;12(8):e1005782. doi: 10.1371/journal.ppat.1005782 (PMC4986965; doi:10.1371/journal.ppat.1005782)

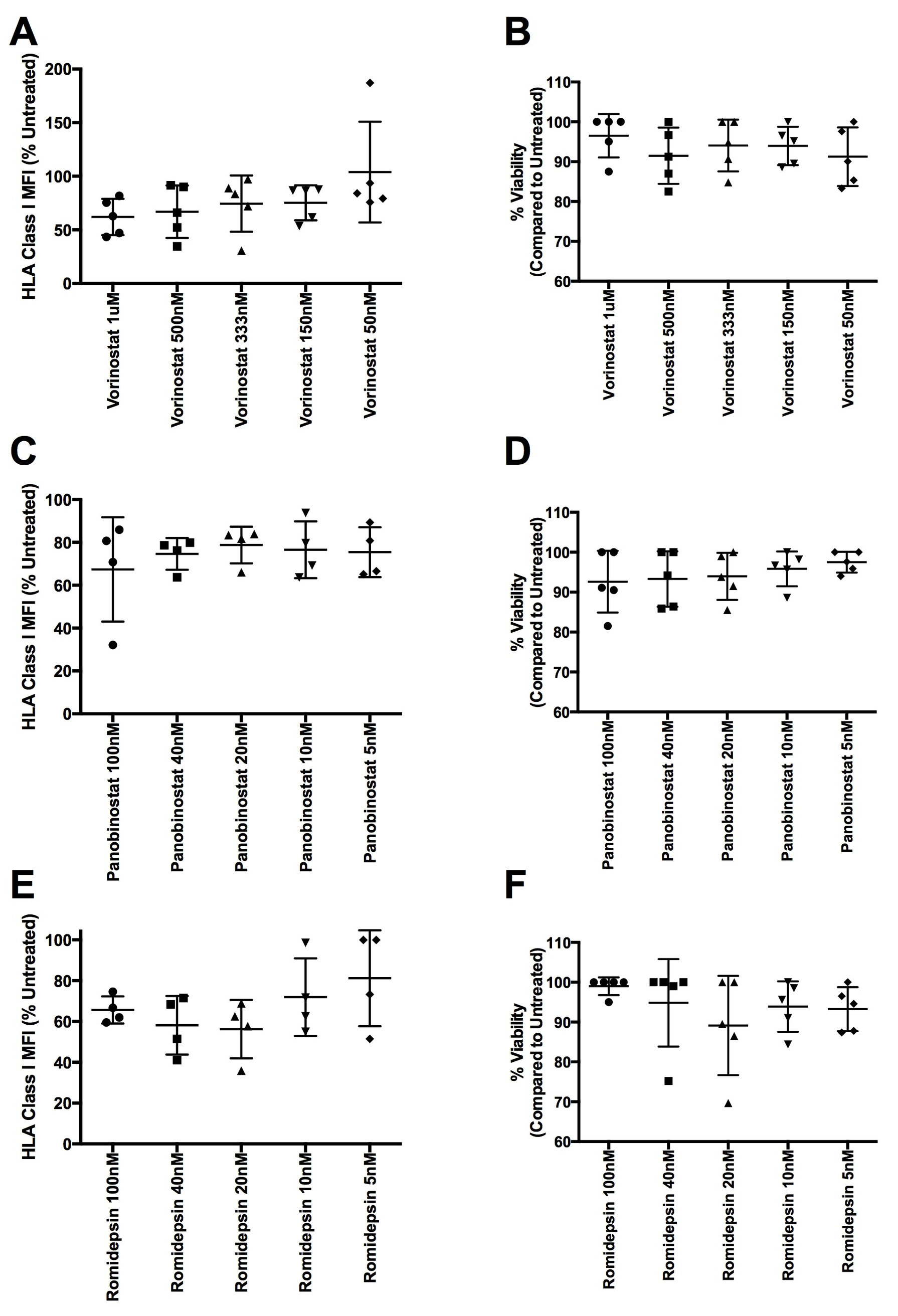

Supplement: S1 Fig — Uninfected CD4 T cells were treated for 24 hours with the indicated dose of HDACi. The MFI of HLA class I as a percent of untreated controls is shown for a range of doses of vorinostat (A), panobinostat (C), and romidepsin (E). The viability of these cells is shown in B, D, and F respectively (n = 5) (TIF) [file ppat.1005782.s001.tif]

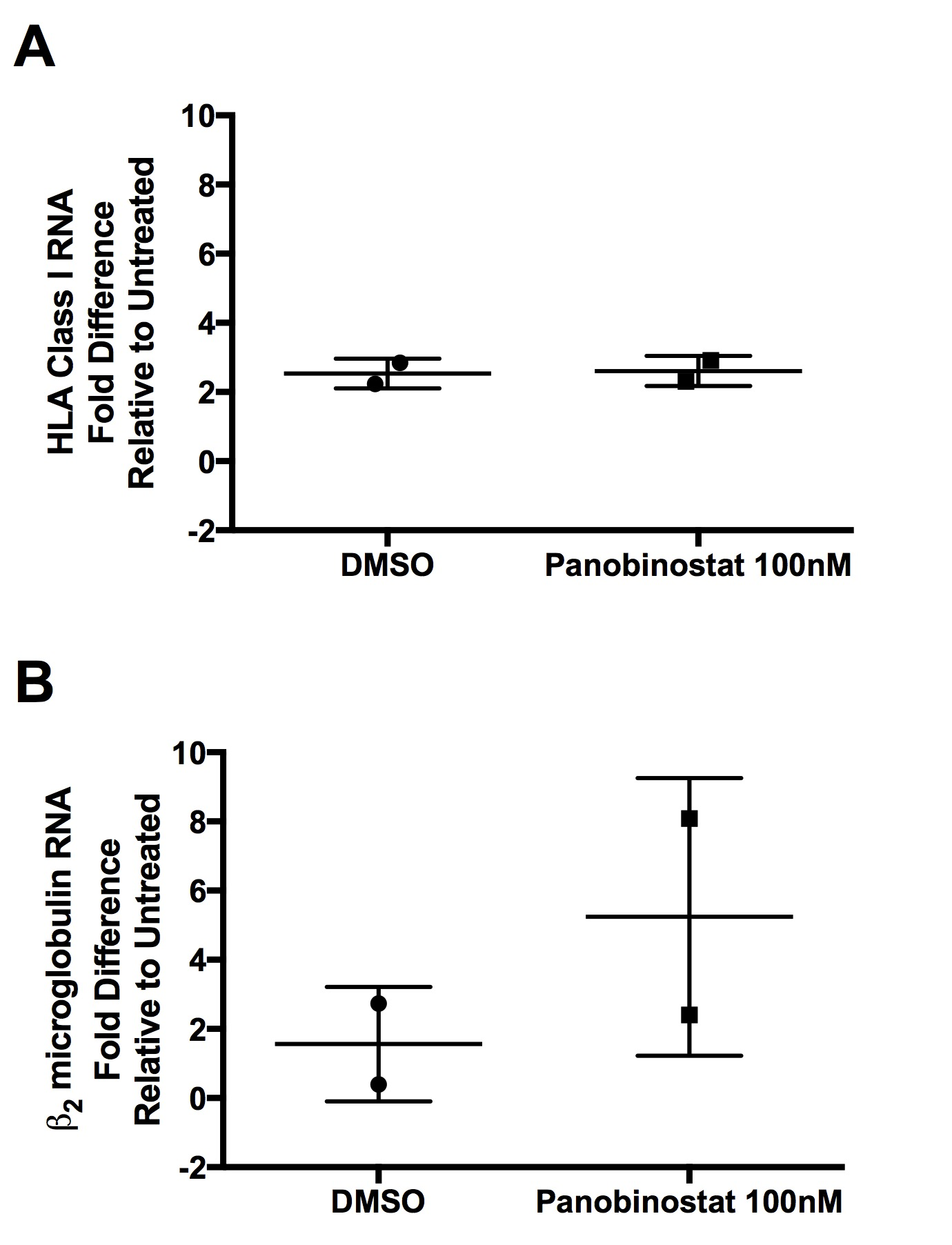

Supplement: S2 Fig — Fold upregulation of HLA class I (A) and β2-microglobulin RNA (B) by qPCR of DMSO and 100nM panobinostat treated samples normalized to untreated controls is shown. All amounts were normalized to 18S copies (n = 2). (TIF) [file ppat.1005782.s002.tif]

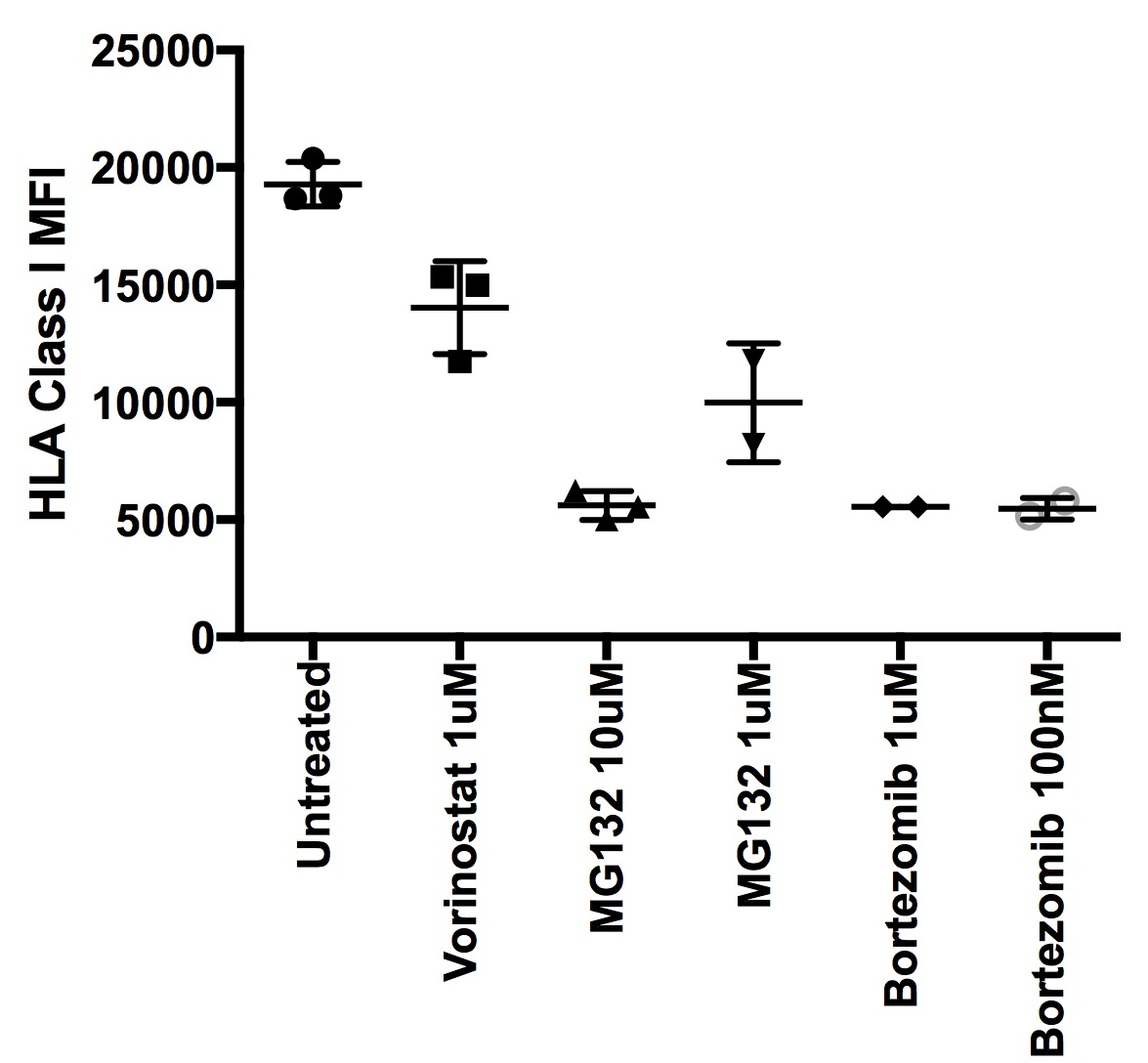

Supplement: S3 Fig — Healthy primary CD4 T cells were cultured in media (untreated) or treated with 1μM vorinostat, 10μM MG132, 1μM MG132, 1μM bortezomib, or 100nM bortezomib. The MFI of HLA class I levels is shown (n = 3 vorinostat, MG132; n = 2 bortezomib) (TIF) [file ppat.1005782.s003.tif]

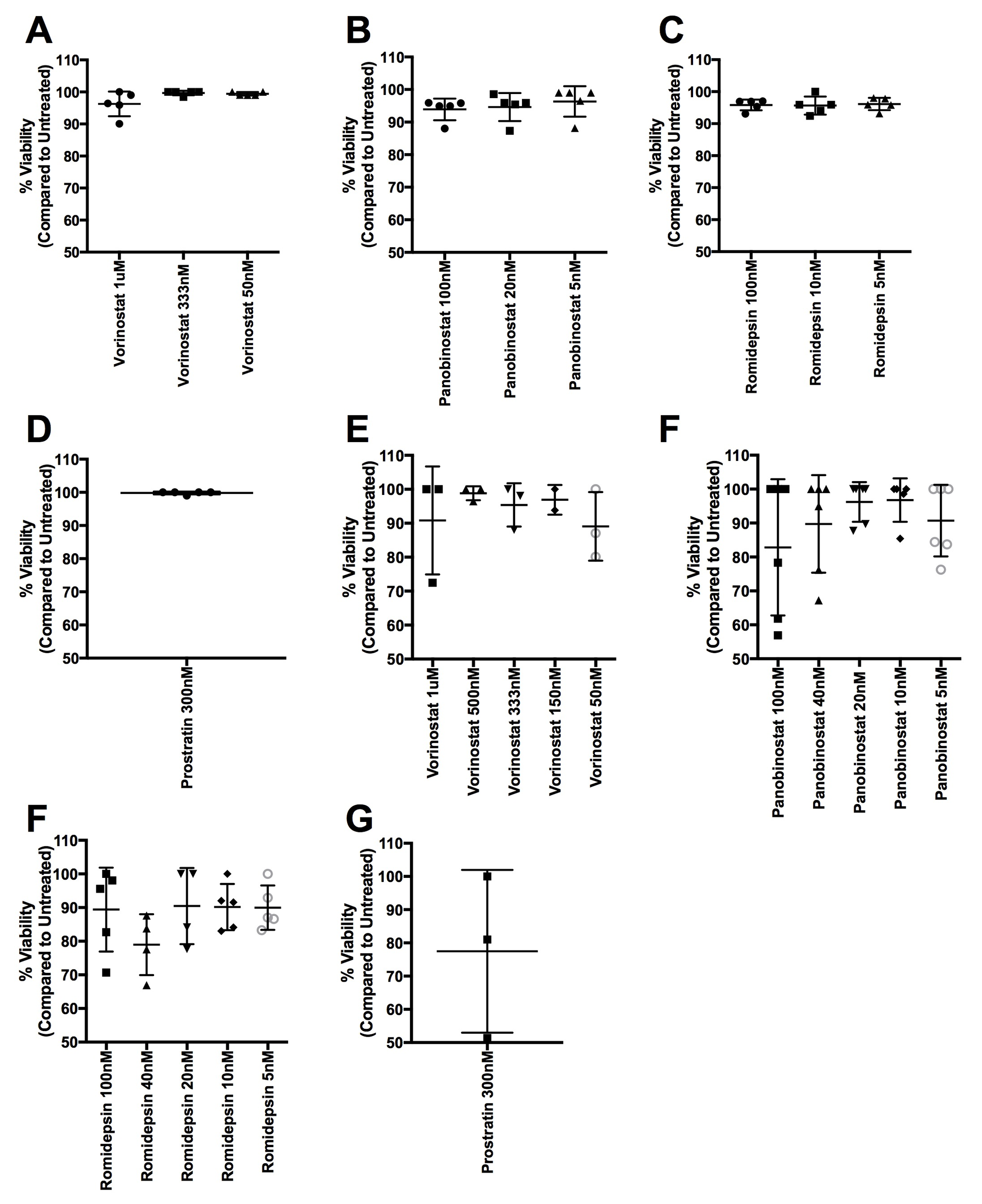

Supplement: S4 Fig — The viability of CD4 T cells from HIV infected patients treated with doses of vorinostat, panobinostat, romidepsin and prostratin is shown in A-D respectively (n = 5). The viability of in vitro infected cells treated with the same drug doses are shown in E-G (n = 3 vorinostat, prostratin; n = 6 panobinostat; n = 5 romidepsin). (TIF) [file ppat.1005782.s004.tif]

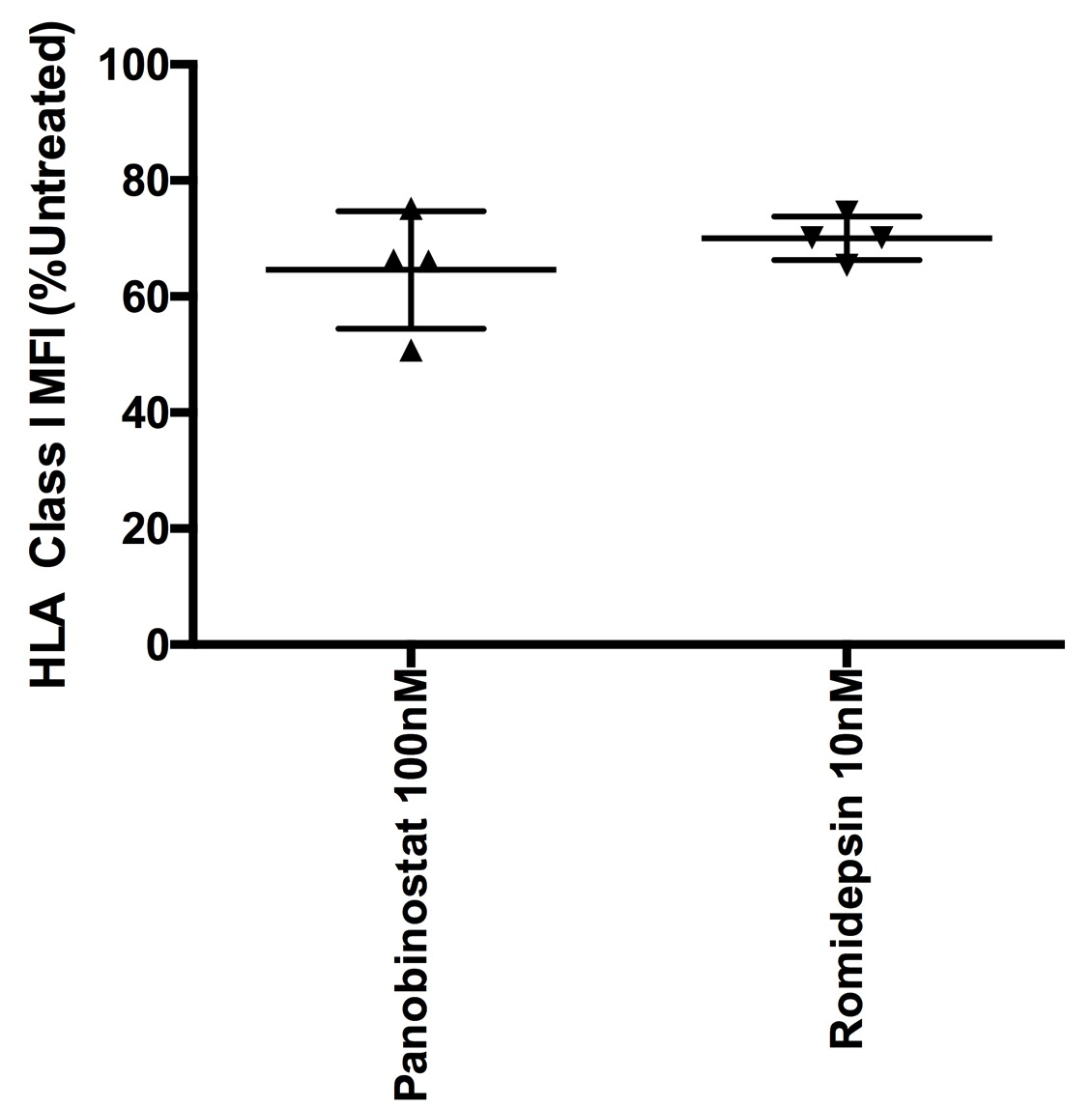

Supplement: S5 Fig — CD4 T cells were spinoculated with HIV-1 LAI for 48 hours and then treated with 100nM panobinostat or 10nM romidepin for 24 hours. HLA Class I levels were then measured and reported as a percent of untreated controls (n = 4). (TIFF) [file ppat.1005782.s005.tiff]

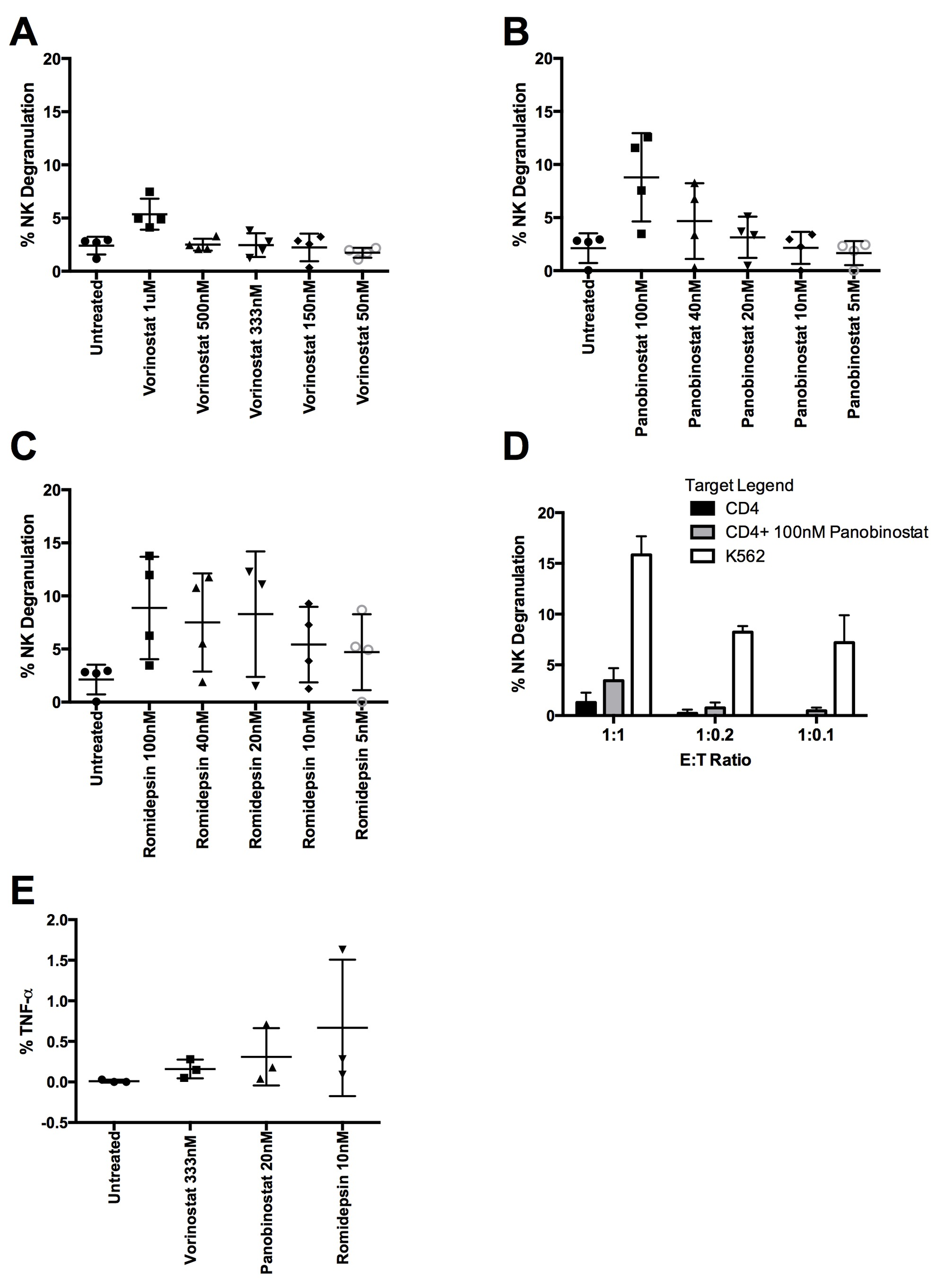

Supplement: S6 Fig — CD4 T cells treated with several doses of vorinostat, panobinostat and romidepsin were co-cultured with NK cells at a 1:1 ratio for 5 hours and CD107a expression was measured in A-C respectively (n = 4). In D, either CD4 T cells treated with or without 100nM panobinostat or untreated K562 cells were co-cultured with NK cells at a 1:1, 1:0.2, or 1:0.1 E:T ratio (n = 3). E) TNF-α production was measured in NK cells co-cultured for 5 hours with cells treated with or without 333nM vorinostat, 20nM panobinostat, or 10nM romidepsin (n = 3). (TIF) [file ppat.1005782.s006.tif]

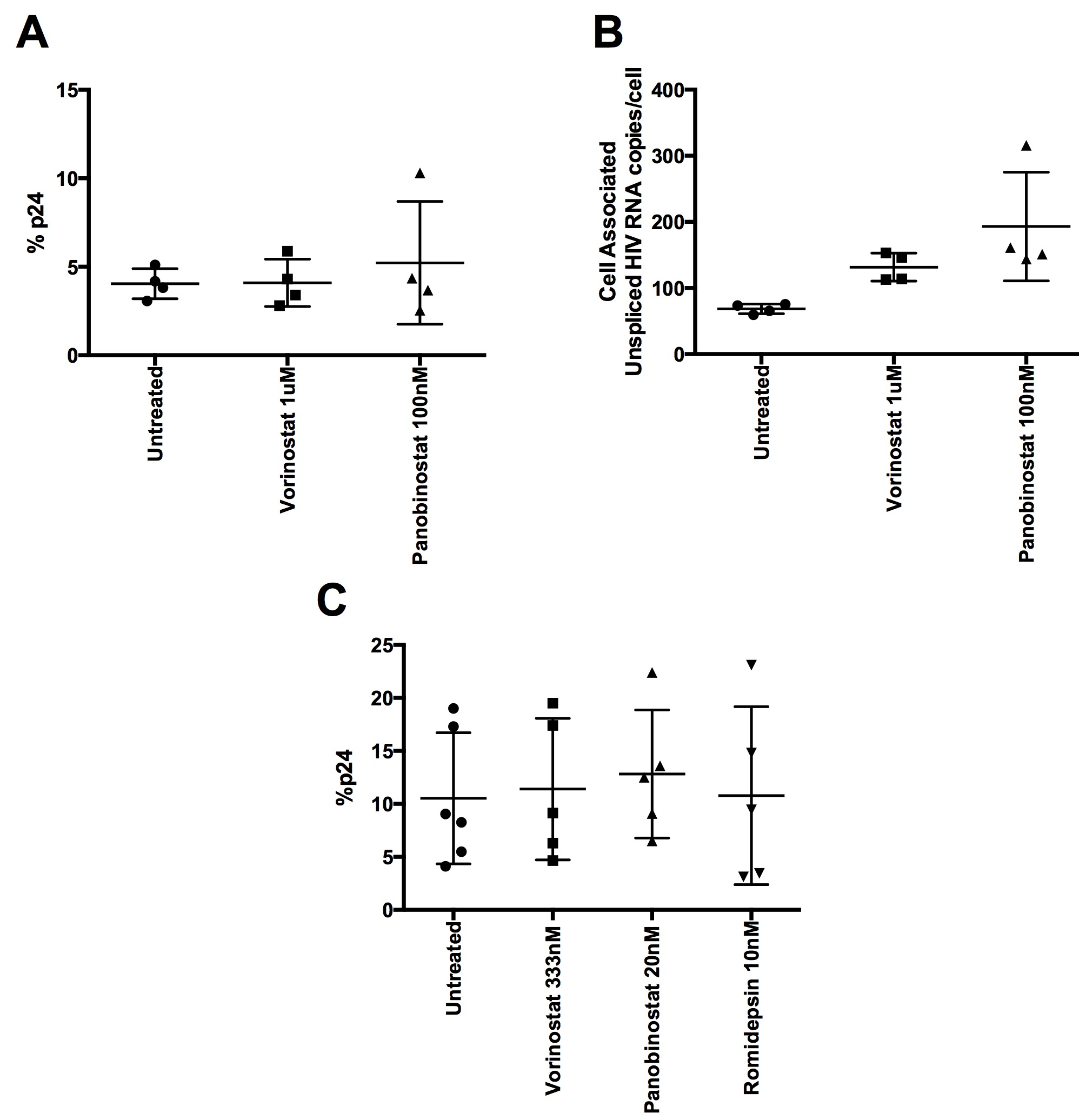

Supplement: S7 Fig — CD4 T cells were infected with LAI for 48 hours after which they were either left in media or treated for 24 hours with 1μM vorinostat or 100nM panobinostat. Intracellular p24 levels (A) and cell- associated unspliced HIV-RNA (B) were measured 72 hours post infection (n = 4). C) Cells were infected as above and treated with 333nM vorinostat, 20nM panobinostat, and 10nM romidepsin for 24 hours. Intracellular p24 levels are shown (n = 5) (TIF) [file ppat.1005782.s007.tif]

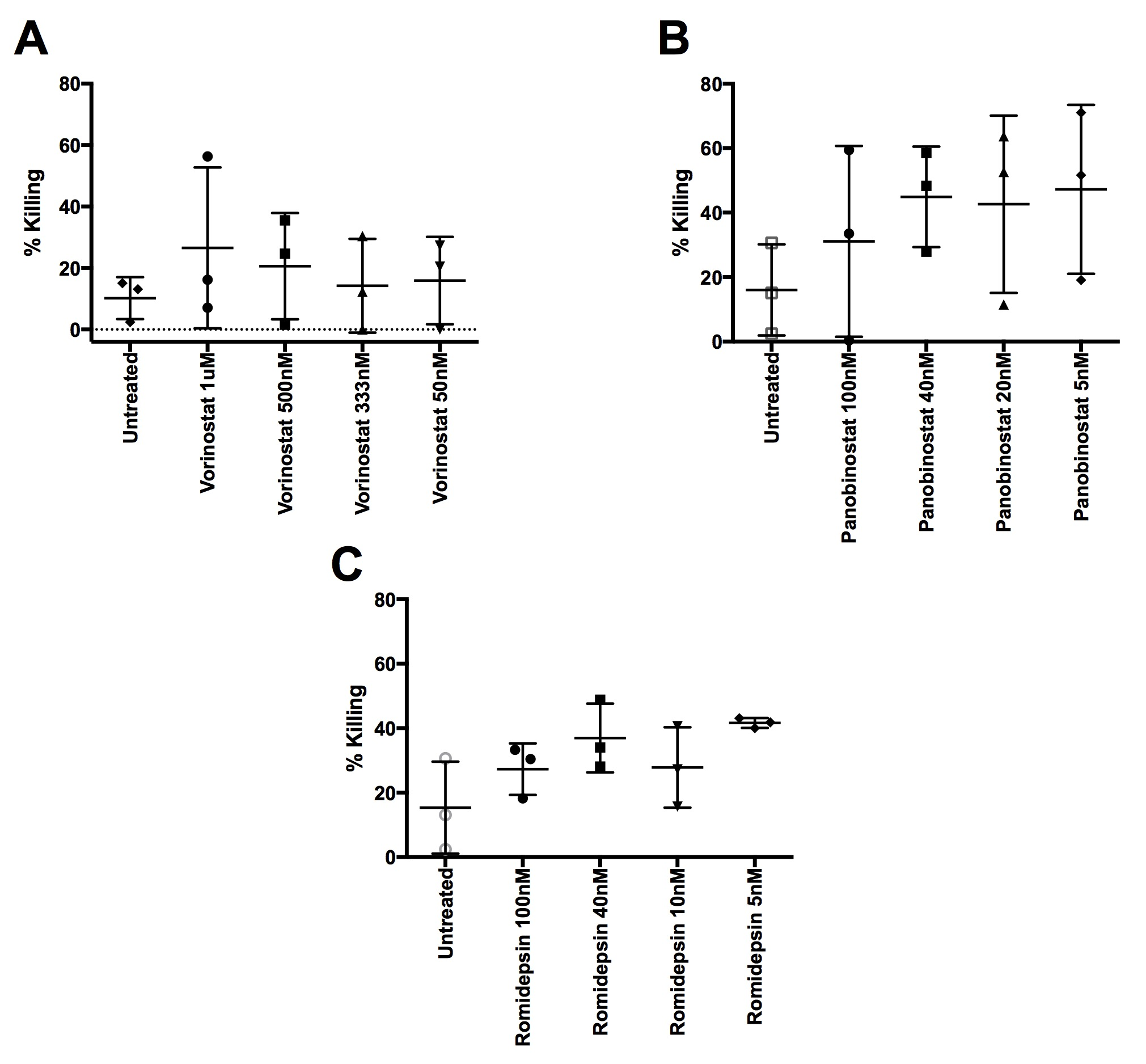

Supplement: S8 Fig — In vitro infected CD4 T cells were treated for 24h with several doses of vorinostat, panobinostat, and romidepsin in A-C respectively and a killing assay based on p24 reduction was performed as in Fig 6 (n = 3). (TIF) [file ppat.1005782.s008.tif]

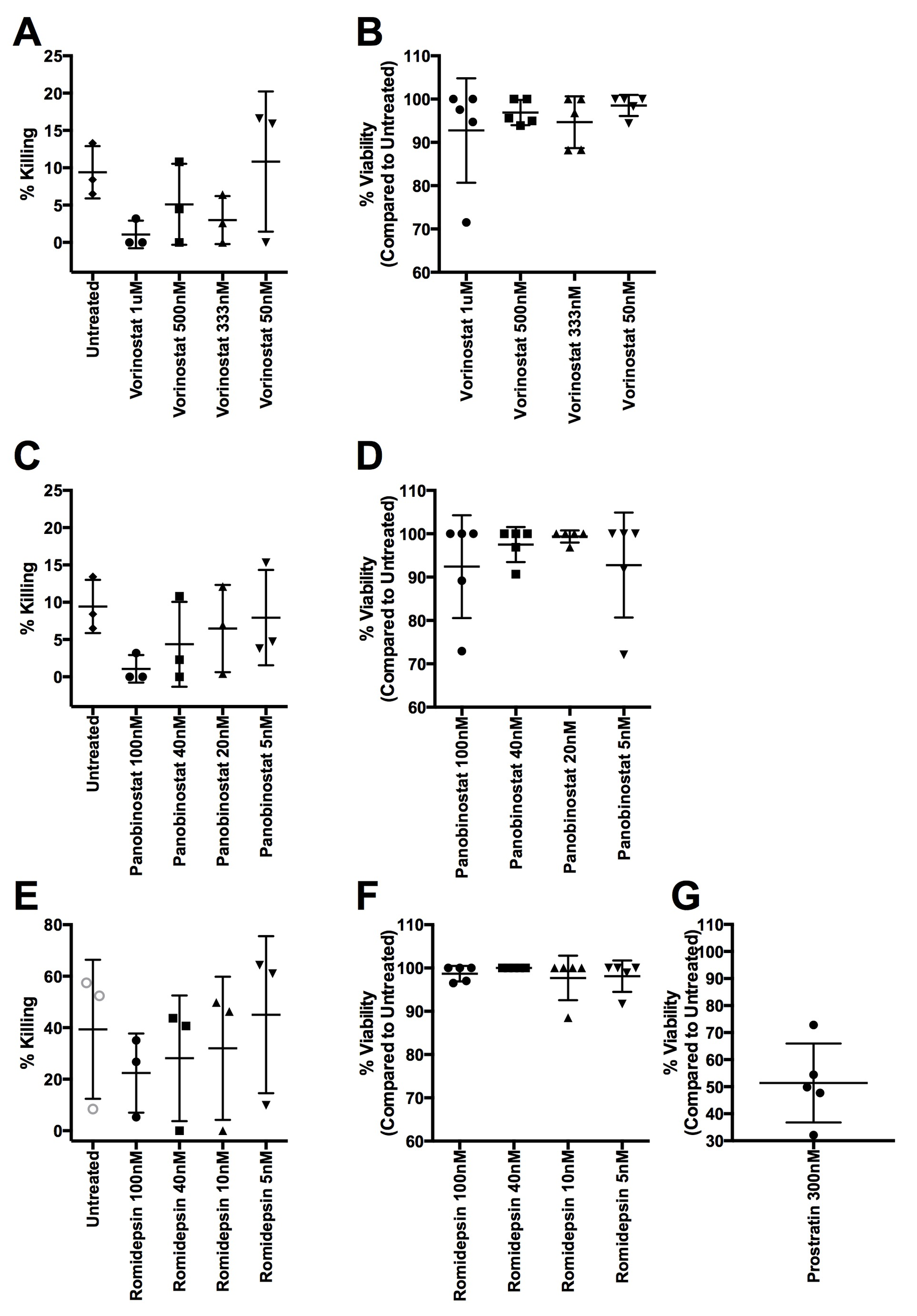

Supplement: S9 Fig — NK cells treated with or without several doses of vorinostat, panobinostat, and romidepsin were co-cultured with infected CD4 T cells and a killing assay based on p24 reduction was performed as described (A, C, and E respectively, n = 3). Viability of the NK cells was measured in B, D, and F for the same HDACi doses (n = 5). In G, viability of NK cells treated with 300nM prostratin was measured (n = 5). (TIF) [file ppat.1005782.s009.tif]

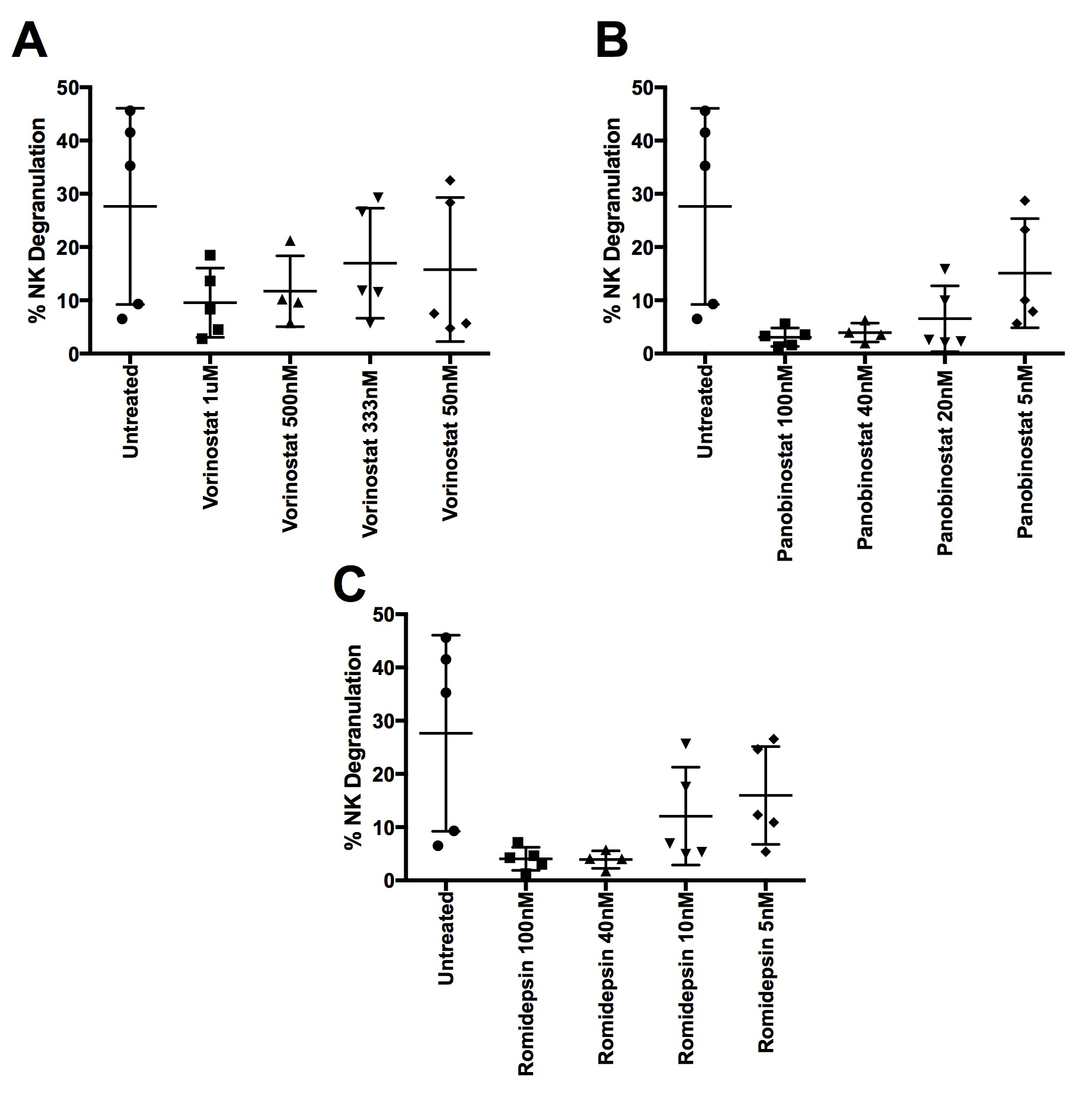

Supplement: S10 Fig — NK cells were treated with various of doses of vorinostat, panobinostat, and romidepsin (A-C respectively) and co-cultured or not with K562 cells for 5 hours at a 1:1 ratio. CD107a expression was measured (n = 5). (TIF) [file ppat.1005782.s010.tif]

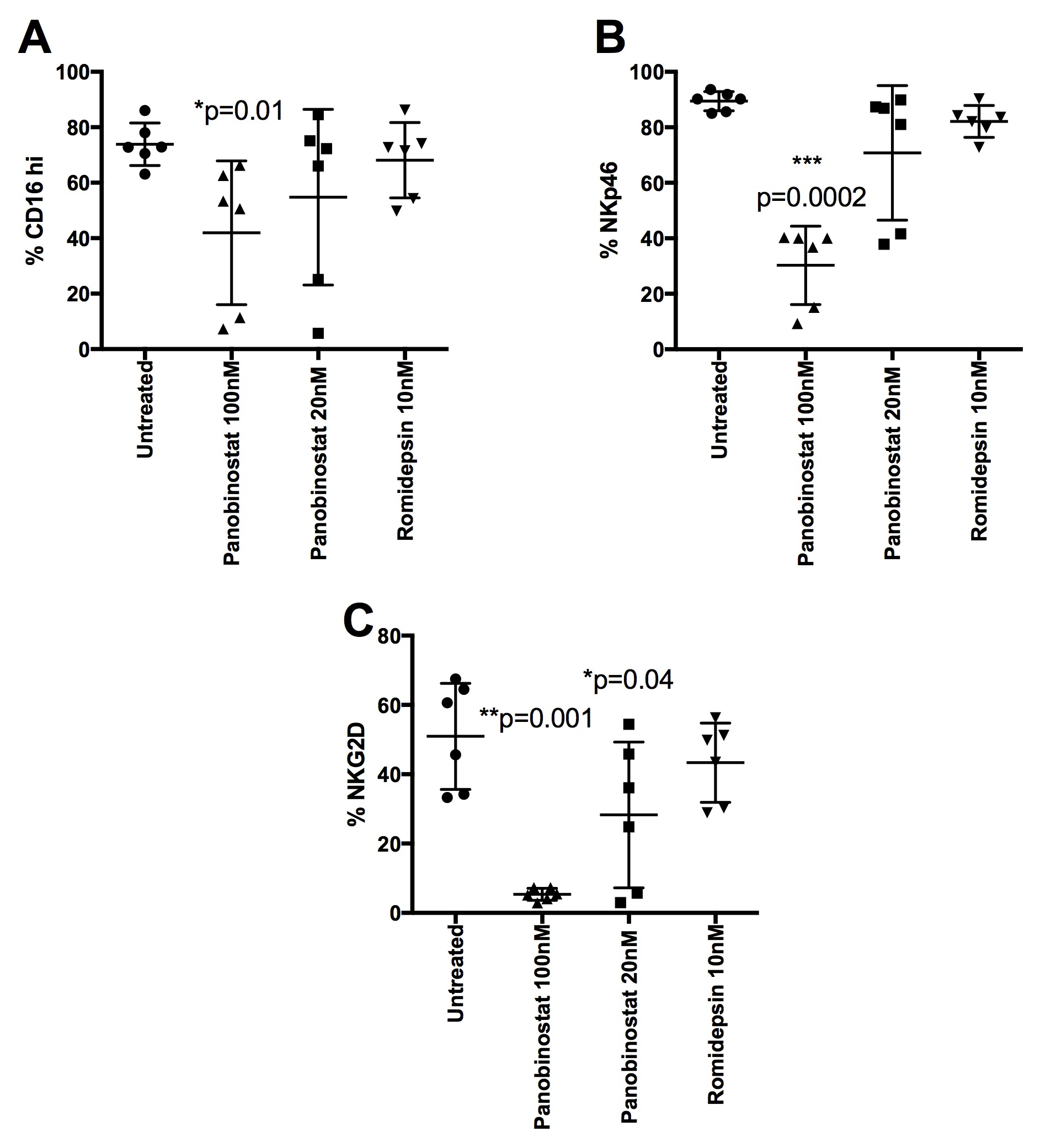

Supplement: S11 Fig — PBMC were cultured with or without 100nM panobinostat, 20nM panobinostat, or 10nM romidepsin for 24h. NK cells were gated using live/dead and lineage markers. The percentage of CD16hi (A) NKp46 (B) and NKG2D (C) expressing cells is shown (n = 6). A Friedman test with Dunn’s test for multiple comparisons was performed. (TIF) [file ppat.1005782.s011.tif]
