# Supplementary material for: Histone Deacetylase Inhibitors Enhance CD4 T Cell Susceptibility to NK Cell Killing but Reduce NK Cell Function
Source: PLoS Pathog. 2016 Aug 16;12(8):e1005782. doi: 10.1371/journal.ppat.1005782 (PMC4986965; doi:10.1371/journal.ppat.1005782)
Supplement: S1 Table — Patient characteristics of the eight HEATHER patients used for HLA Class I measurements. (PDF) [file ppat.1005782.s012.pdf]

ID	Ethnic	Gender	CD4 Count	VL	ART Regimen	Time on ART (months)
ST-015	Caucasian	Male	659	<20	emtricitabine, tenofovir, darunavir, ritonavir	24
ST-033	Caucasian	Male	791	<20	abacavir, dolutegravir, lamivudine	5
SM-47	Caucasian	Male	750	<20	efavirenz, emtricitabine, tenofovir	32
SM-49	Caucasian	Male	1160	30	efavirenz, emtricitabine, tenofovir	37
SM-73	Asian	Male	1039	<40	abacavir, lamivudine, darunavir, ritonovir,	15
SM-130	Caucasian	Male	550	<20	efavirenz, emtricitabine, tenofovir	6
SM-138	Greek	Male	475	53	emtricitabine, tenofovir, raltegravir	1
SM-140	Caucasian	Male	393	<40	emtricitabine, tenofovir, darunavir, ritonavir	5
